# Strain regulation retards natural operation decay of perovskite solar cells

**DOI:** 10.1038/s41586-024-08161-x

**Published:** 2024-10-14

**Authors:** Yunxiu Shen, Tiankai Zhang, Guiying Xu, Julian A. Steele, Xiankai Chen, Weijie Chen, Guanhaojie Zheng, Jiajia Li, Boyu Guo, Heyi Yang, Yeyong Wu, Xia Lin, Thamraa Alshahrani, Wanjian Yin, Jian Zhu, Feng Wang, Aram Amassian, Xingyu Gao, Xiaohong Zhang, Feng Gao, Yaowen Li, Yongfang Li

**Affiliations:** 1 Laboratory of Advanced Optoelectronic Materials, Suzhou Key Laboratory of Novel Semiconductor-optoelectronics Materials and Devices, College of Chemistry, Chemical Engineering and Materials Science, Soochow University, Suzhou, China; 2 Department of Physics, Chemistry and Biology (IFM), Linköping University, Linköping, Sweden; 3 Australian Institute for Bioengineering and Nanotechnology and School of Mathematics and Physics, The University of Queensland, Brisbane, Queensland Australia; 4 Institute of Functional Nano and Soft Materials (FUNSOM), Soochow University, Suzhou, China; 5 Shanghai Synchrotron Radiation Facility (SSRF), Zhangjiang Lab, Shanghai Advanced Research Institute, Chinese Academy of Sciences, Shanghai, China; 6 State and Local Joint Engineering Laboratory for Novel Functional Polymeric Materials, Jiangsu Key Laboratory of Advanced Functional Polymer Design and Application, College of Chemistry, Chemical Engineering and Materials Science, Soochow University, Suzhou, China; 7 Department of Materials Science and Engineering and Organic and Carbon Electronics Laboratories (ORaCEL), North Carolina State University, Raleigh, NC USA; 8 Department of Physics, College of Science, Princess Nourah bint Abdulrahman University, Riyadh, Saudi Arabia; 9 College of Energy, Soochow Institute for Energy and Materials InnovationS (SIEMIS), Soochow University, Suzhou, China; 10 Jiangsu Key Laboratory of Advanced Negative Carbon Technologies, Soochow University, Suzhou, China; 11 Beijing National Laboratory for Molecular Sciences; CAS Key Laboratory of Organic Solids, Institute of Chemistry, Chinese Academy of Sciences, Beijing, China

**Keywords:** Solar cells, Solar cells

## Abstract

Perovskite solar cells (pero-SCs) have undergone rapid development in the past decade. However, there is still a lack of systematic studies investigating whether the empirical rules of working lifetime assessment used for silicon solar cells can be applied to pero-SCs. It is believed that pero-SCs show enhanced stability under day/night cycling owing to the reported self-healing effect in the dark^1,2^. Here we find that the degradation of highly efficient FAPbI_3_ pero-SCs is much faster under a natural day/night cycling mode, bringing into question the widely accepted approach to estimate the operational lifetime of pero-SCs based on continuous-mode testing. We reveal the key factor to be the lattice strain caused by thermal expansion and shrinking of the perovskite during operation, an effect that gradually relaxes under the continuous-illumination mode but cycles synchronously under the cycling mode^3,4^. The periodic lattice strain under the cycling mode results in deep trap accumulation and chemical degradation during operation, decreasing the ion-migration potential and hence the device lifetime^5^. We introduce phenylselenenyl chloride to regulate the perovskite lattice strain during day/night cycling, achieving a certified efficiency of 26.3 per cent and a 10-fold improvement in the time required to reach 80% of peak efficiency (*T*_80_) under the cycling mode after the modification.

## Abstract (web summary)

Perovskite solar cells degrade quickly under natural day/night cycling, compared with continuous illumination, owing to periodic lattice strain during cycling; the lattice strain can be regulated by adding phenylselenenyl chloride.

## Main

State-of-the-art stability protocols (the International Summit on Organic Photovoltaic Stability (ISOS) protocols) have been well developed based on the experience gained from commercialized silicon solar cells. However, although solar panels work under natural day/night cycling in practical operation, most accelerated stress test protocols (such as ISOS-L or ISOS-LC) use continuous illumination to estimate the panel lifetime^6–8^. For perovskite solar cells (pero-SCs), the first issue that might challenge the validity of lifetime estimation by following the established silicon protocols under continuous-illumination mode is the ionic defects in perovskites, which induce a ‘fatigue’ behaviour under the day/night cycling mode, and performance recovery after a period of rest in darkness^9–11^.

In addition, although the ionic defects could potentially be minimized by rational passivation, another intrinsic feature of perovskites, a soft crystal lattice, also makes the continuous mode significantly different from the cycling mode in pero-SCs^11–13^. Temperature variation in the natural day/night cycling process results in lattice expansion and shrinking in soft perovskites, which heavily affects the performance of pero-SCs^3–5,14–16^. However, previous studies on the stability of pero-SCs under the day/night cycling mode mostly fixed the operation temperature at room temperature (as well as the ISOS-LC protocols), which neglects the temperature-induced effects (Supplementary Table 1). A very recent study revealed that the stability of pero-SCs could be quite different under natural day/night cycling mode compared with the constant-illumination mode through a detailed tracking for more than 2 years^17^. As such, it is crucial to develop a thorough understanding of the lifetime difference between the natural cycling mode and the continuous mode.

Here we investigate the degradation process of pero-SCs under both the continuous-illumination mode (denoted as continuous mode) and natural day/night cycling mode considering both illumination and temperature fluctuations (denoted as cycling mode), and find that pero-SCs degrade faster under the cycling mode. The temperature variation in the cycling mode induces cycled lattice volume change, resulting in periodic lattice strain, whereas the continuous mode gradually releases the lattice strain. During operation, the strained lattice leads to the accumulation of deep traps that are difficult to heal in the dark, greatly accelerating the device degradation. To eliminate faster degradation of perovskites induced by lattice strain in the cycling mode, a phenylselenium compound that has strong coordination to Pb^2+^ ions and a large steric effect is designed to modify the crystallization and anchor to the grain boundaries. As a result, the optimized device achieves a promising certified power conversion efficiency (PCE) of 26.3%. Importantly, the time required to reach 80% of peak efficiency (*T*_80_  lifetime) under the cycling mode was extended tenfold compared with the continuous mode, showing that regulating the lattice strain maximizes the device lifetime under a simulated natural day/night cycling mode. This work uncovers the largely neglected point that degradation under daily cycling is heavily accelerated by periodic lattice strain evolving from lattice expansion and shrinking compared with the continuous mode. In addition, it highlights the importance of establishing accelerated ageing protocols for estimating the pero-SC lifetime under real working conditions, paving the way for their successful commercialization.

## Faster PCE decay under cycling

**Fig. 1:**
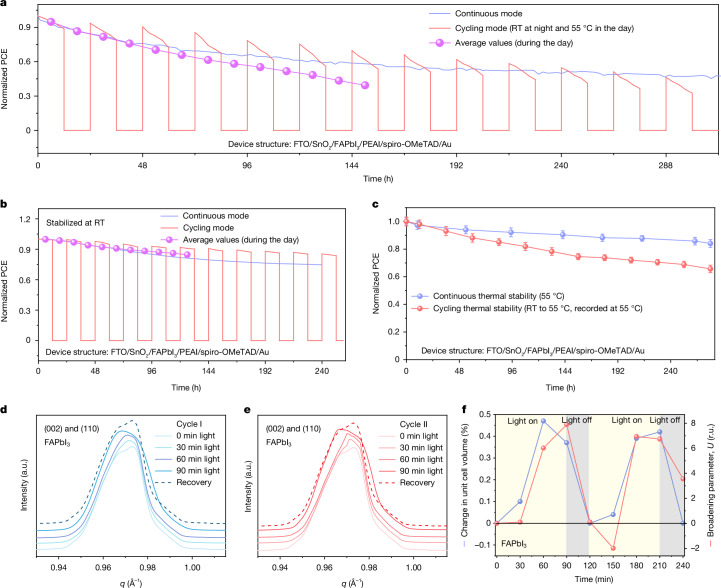
Faster PCE decay and cycled lattice strain in the cycling mode. **a**, Stability of the pero-SCs based on FAPbI_3_ working in the continuous and cycling modes. The purple line represents the average value for each cycling mode with illumination time. **b**, Stability of the pero-SCs based on FAPbI_3_ working in the continuous and cycling modes. The temperature of the device was fixed at room temperature (RT; about 25 °C). **c**, Stability of the pero-SCs based on FAPbI_3_ working in the continuous thermal and cycled thermal (12 h at room temperature and 12 h at about 55 °C) ageing modes. Error bars represent the standard deviation of five devices for each condition. **d**,**e**, Integrated profiles (out of plane) obtained from in situ GIWAXS maps for the FAPbI_3_ perovskite film deposited on a silicon wafer substrate. The film was illuminated for 0 to 90 min and measured at 30-min intervals for the two cycles (**d**, the first cycle; **e**, the second cycle), and the recovery spectra were obtained from the film kept in dark for 30 min. **f**, Evolution of the unit cell volume and the peak broadening parameters for the FAPbI_3_ samples during in situ GIWAXS measurements. r.u., relative units. Source Data

We used pero-SCs based on the structure of SnO_2_/formamidinium lead triiodide (FAPbI_3_)/phenylethylammonium iodide (PEAI)/2,2′,7,7′-tetrakis(N,N-di-p-methoxyphenyl-amine)9,9′-spirobifluorene (spiro-OMeTAD) as an example and put our main effort in to exploring their PCE degradation under continuous and cycling modes^7^. The detailed ageing conditions and the initial performance of the devices are summarized in Extended Data Table 1 and Supplementary Table 2, respectively. Owing to the heat effect from infrared radiation^4^, the temperature gradually increases to about 55 °C under the continuous mode and cycles between about 25 °C (room temperature) and about 55 °C under the cycling mode (Supplementary Fig. 1). As shown in Fig. 1a, in the continuous mode, the device shows a continuously decreasing degradation rate with increasing illumination time, with a PCE decay rate of about 0.5% h^−1^ in the first 12 h, which gradually retards to about 0.25% h^−1^ after around 120 h. In the cycling mode, consistent with previous reports, the PCE is found to partially recover during the dark period, ascribed to the defects self-healing process^18^. However, after the recovery, the PCE decays at a slightly higher rate (for example, with a PCE decay rate of about 0.75% h^−1^ in the second cycle, compared with about 0.5% h^−1^ in the first cycle). This faster degradation rate in the cycling mode results in more severe overall degradation of pero-SCs than that in the continuous mode after only two cycles (with the same illumination time), which has rarely been reported previously. As a result, after continuous illumination for 156 h, the pero-SCs based on FAPbI_3_ can maintain about 57% of their initial efficiency, whereas only about 39% of the initial efficiency is retained after the pero-SCs have aged for 13 day/night cycles with the same illumination time (Supplementary Fig. 2).

**Extended Data Fig. 1:**
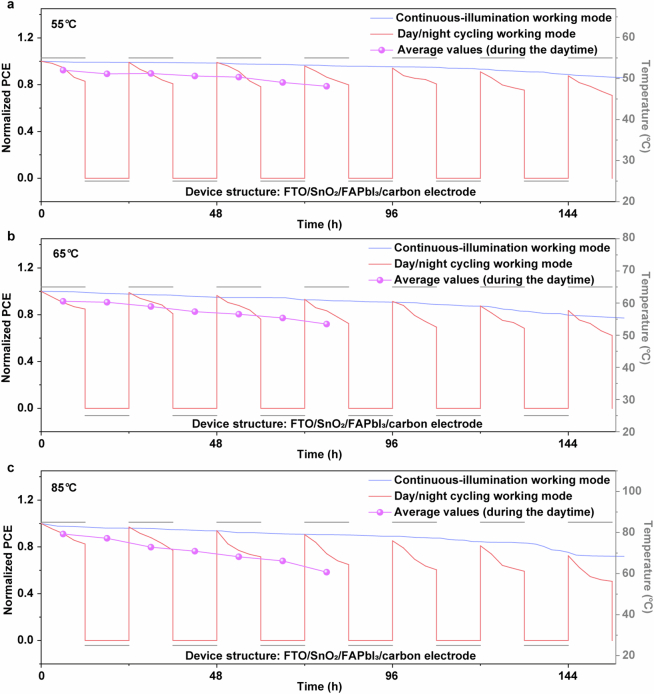
Faster PCE decay of pero-SCs based on FAPbI_3_ in the day/night cycling working mode. **a-c**, PCE tracking of the pero-SCs based on FAPbI_3_/carbon electrode working in the continuous-illumination and day/night cycling modes (**a**, day period: ~55 °C; **b**, day period: ~65 °C; **c**, day period: ~85 °C). Source Data

**Extended Data Fig. 2:**
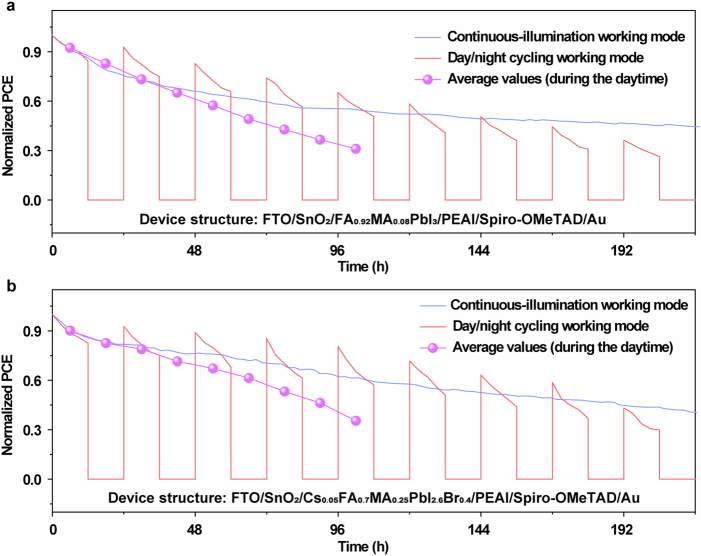
Faster PCE decay of pero-SCs based on other perovskite active layers in the day/night cycling working mode. **a,b**, PCE tracking of the pero-SCs based on **a**, FA_0.92_MA_0.08_PbI_3_ and **b**, Cs_0.05_FA_0.7_MA_0.25_PbI_2.6_Br_0.4_ working in the continuous-illumination and day/night cycling modes. Source Data

Subsequently, we explored the dominant factor for the faster decay of pero-SCs under the cycling mode from the perspective of the device structure. As shown in Supplementary Fig. 3, the devices with or without any surface passivation (for example, PEAI or octylammonium iodide) showed severe degradation under the cycling mode, although surface passivation can alleviate the degradation to a certain extent. This indicates that surface passivation is not the key factor leading to the accelerated decay of pero-SCs under the cycling mode. Then we compared the device degradation under the continuous and cycling modes using a simple device structure of SnO_2_/FAPbI_3_/carbon electrode, which excludes the unstable factors from the degradation of the hole transport layers (HTLs) or metal electrode corrosion induced by ion migration. As shown in Extended Data Fig. 1, all the devices showed severe degradation under the cycling mode, confirming that the accelerated decay is mainly caused by the perovskite active layer. This severe degradation of pero-SCs under the cycling mode is also observed in two other perovskite systems, FA_0.92_MA_0.08_PbI_3_ and Cs_0.05_FA_0.7_MA_0.25_PbI_2.6_Br_0.4_ (Extended Data Fig. 2), indicating that this abnormal faster degradation under day/night cycling might be a general issue for perovskites with different compositions.

As the cycling mode involves cycled illumination and temperature fluctuation, we decouple these two effects to investigate their respective impact on the device lifetime. Interestingly, if we compare the device PCE decay process under the continuous and the cycled illumination with a fixed temperature of about 25 °C (ISOS-L-1 and ISOS-LC-1 protocols), the PCE decay rate under the cycling mode is slightly lower (Fig. 1b), which is consistent with previous reports^6^. In other words, cycled illumination with a fixed temperature of about 25 °C does not lead to faster degradation. However, by comparing the PCE decay under the continuous (about 55 °C) and the cycled (room temperature to about 55 °C) thermal ageing (both without illumination), we find more severe degradation under the cycled thermal ageing (about 66% PCE remains after cycled thermal ageing whereas about 90% PCE is maintained after continuous thermal ageing with the same thermal ageing time; Fig. 1c). In addition, by systematically comparing the device (SnO_2_/FAPbI_3_/carbon electrode) decay at different temperatures (about 55 °C, 65 °C and 85 °C), we found more severe degradation of pero-SCs under the cycling mode (Extended Data Fig. 1), indicating that severe degradation is independent of the specific temperature during the day period and determined by temperature fluctuations.

We now understand that the cycled temperature has an important role in the faster PCE decay under the cycling mode. It is well acknowledged that temperature change can result in crystal lattice distortion and symmetry change, especially for FAPbI_3_ which has a large thermal expansion coefficient (Supplementary Figs. 4 and 5, as mentioned in Supplementary Note 1). We thus turn to crystallographic analysis to determine the faster degradation mechanism in the cycling mode.

## Crystallographic analysis under cycling

**Extended Data Fig. 3:**
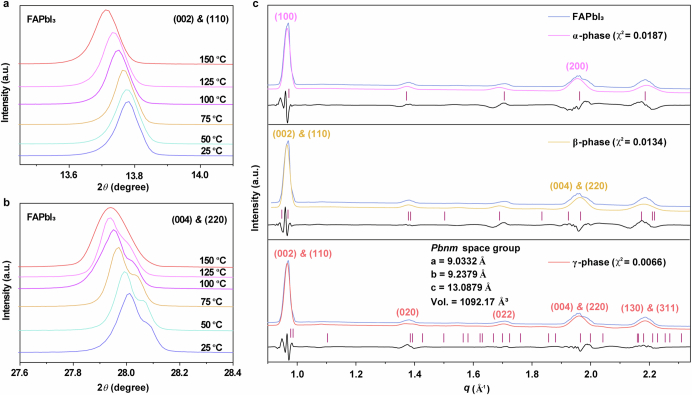
The orthorhombic phase for FAPbI_3_ at RT. **a,b**, Temperature-dependent XRD patterns (cooling process) of perovskite films based on FAPbI_3_ (focused on (002)/(110) and (004)/(220) diffractions, respectively). **c**, Comparison of structural refinements made using cubic (α-phase), tetragonal (β-phase) and orthorhombic (γ-phase) perovskite structures. The goodness of fit values (χ^2^) is inset, confirming that the best fit is made with a γ-phase structure at RT. Source Data

At room temperature before illumination, the perovskite stays in an orthorhombic phase based on angle-dependent grazing-incidence wide-angle X-ray scattering (GIWAXS) measurements (Extended Data Fig. 3c). The characteristic diffraction peaks become more asymmetric when the X-ray incident angle goes from above the critical angle (about 0.15°) to below that angle, indicating that the octahedral distortion is more significant from the bulk to the surface of the perovskite layer (Supplementary Fig. 6). The gradually increased octahedral distortion shows that the lattice shrinking is restricted owing to the interaction between perovskites and the metal-oxide electron transport layer, implying the generation of tensile strain across the perovskite layer^14,16^.

To reveal the crystallographic evolution of the perovskite layer during the cycling mode, we performed in situ synchrotron GIWAXS measurements under cycled light illumination and temperature fluctuations that mimic the cycling mode (Supplementary Fig. 7). With increasing illumination time and increasing temperature from room temperature to about 55 °C, the characteristic diffraction peak of the perovskite gradually shifts to a smaller scattering vector *q* value and then stabilizes (Fig. 1d,e). The more symmetric cubic-like lattice in the light period is also confirmed by a detailed characteristic diffraction peak shape analysis (Supplementary Fig. 8). Upon cooling the sample to room temperature, the *q* value returns to the original state and the lattice shrinks back to the orthorhombic phase with PbI_6_ octahedral distortion, ascribed to the lead-halide sublattice bonds shrinking in length more slowly than the cation ionic sublattice during the cooling process^19^. As revealed by the calculation of lattice constants and peak broadening from in situ GIWAXS results, the perovskite lattice undergoes a synchronous symmetry increase and a relatively large lattice volume evolution (0 to about 0.48%) along with the cycling (Fig. 1f). As a result, the tensile strain is released with the lattice expansion under illumination and heating, and reforms during the dark period.

The periodic lattice strain in the cycling mode results in significant film stress variation. Under the daytime operation, the film has a tensile stress of about 45 MPa and then increases to about 75 MPa in the dark^20^ (Supplementary Fig. 9, as mentioned in Supplementary Note 2). The significant film stress variation in the cycling mode results in cracks in the aged pero-SCs (Supplementary Fig. 10), implying poor interface and increased defect densities.

## Defect evolution under cycling

**Fig. 2:**
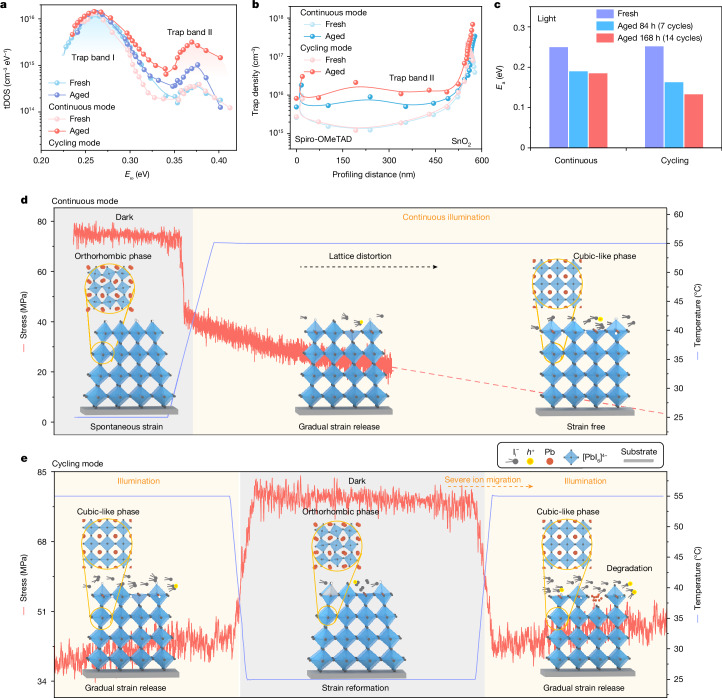
Defect evolution, ion-migration dynamics and stress evolution in the cycling mode. **a**, The tDOS spectra of the pero-SCs before and after ageing under the continuous mode (168 h) and the cycling mode (14 cycles). *E*_ω_ represents the energetic distribution, as mentioned in Supplementary Note 3. **b**, Spatial distribution of the trap densities of trap band II in the pero-SCs before and after ageing under the continuous mode (168 h) and the cycling mode (14 cycles). SnO_2_ and spiro-OMeTAD in the graphs indicate the locations that are close to the SnO_2_ or spiro-OMeTAD layers of the device. **c**, *E*_a_ of the FAPbI_3_ device before and after ageing in the continuous mode and the cycling mode. **d**,**e**, Illustration of the degradation mechanism for the pero-SCs in the continuous mode and the cycling mode. Perovskite film stress was calculated from curvature measurements, as mentioned in Supplementary Note 2. Source Data

It is known that the performance of pero-SCs is heavily affected by the defect density^21,22^; thus, we investigated the evolution of defects in the different working modes (as mentioned in Supplementary Note 3). From thermal admittance spectroscopy (Supplementary Fig. 11), the trap density of states (tDOS) and their energetic distribution are mapped (Fig. 2a). This shows that the aged devices (under both continuous and cycling modes) have the same two trap bands compared with the fresh sample: one centred at about 0.27 eV (shallow trap region: trap band I) and another centred at about 0.37 eV (deep trap region: trap band II)^23^. Although the intensity of trap band I is comparable in both ageing conditions (continuous and cycling modes), the intensity increase of trap band II in the cycling mode is much higher than that in the continuous mode, indicating that the lattice strain cycling is likely to induce more deep trap states and more lead iodide (PbI_2_) generation (Supplementary Fig. 12). Drive-level capacitance profiling results (Fig. 2b and Supplementary Fig. 13) further reveal that the defects density of trap band II increases more notably across the perovskite/transport layer interfaces under the cycling mode compared with the continuous mode.

Along with accumulated defects, we also found continuously decreasing ion motion activation energy (*E*_a_) from 0.25 eV to 0.13 eV after 14 cycles (illumination time of 168 h), whereas *E*_a_ is almost constant at 0.19 eV during continuous ageing (Fig. 2c and Supplementary Fig. 14). The continuously decreasing *E*_a_ with increasing number of cycles reveals an aggravated ion migration during the cycling mode, which leads to more severe degradation (Supplementary Fig. 15). The deep defects accumulation and reduced ion-migration activation energy in the cycling mode can be ascribed to the lattice strain reformation after each dark period^5,16^.

The aforementioned characterizations rationalize the much shortened lifetime of the pero-SCs under the cycling mode. Upon light illumination with temperature increase (for both working modes), the PCE undergoes a fast decay because of the lattice strain in the orthorhombic-phase perovskite. The lattice strain together with light- and heat-induced ion migration leads to the formation of deep-level defects^5,24^. After this fast decay, the two working modes show different decay trends. In the continuous mode (Fig. 2d), illumination and the operational temperature (about 55 °C) gradually release the lattice strain in the device with perovskite lattice expansion towards a higher symmetric configuration, retarding the generation of defects and PCE decay. However, in the cycling mode (Fig. 2e), the lattice shrinks and returns to the asymmetric orthorhombic phase in the dark period at room temperature, which leads to lattice strain reformation. As a result, the shrunken lattice in the next day period continuously induces deep traps, which can hardly be self-healed. The accumulation of deep traps in perovskites speeds up the device degradation with increasing day/night cycles^14^.

## Strain regulation by phenylselenenyl chloride

As such, mitigating the perovskite lattice strain is vital to increase the stability under the natural cycling mode. There are two major parts that contribute to the lattice strain under cycling. One is the lattice phase evolution from the orthorhombic phase at room temperature towards a higher-symmetry phase at elevated temperature; the other is the thermal- and illumination-induced lattice expansion^25,26^. Considering that coordinative additives can greatly affect the crystal orientation, phase and thus thermal expansion behaviour without affecting the composition^27–29^, we were motivated to make use of coordinative additives to promote the stabilization of FAPbI_3_ in a higher-symmetry phase and allow for a reduced lattice volume change through the anchoring effect.

**Extended Data Fig. 4:**
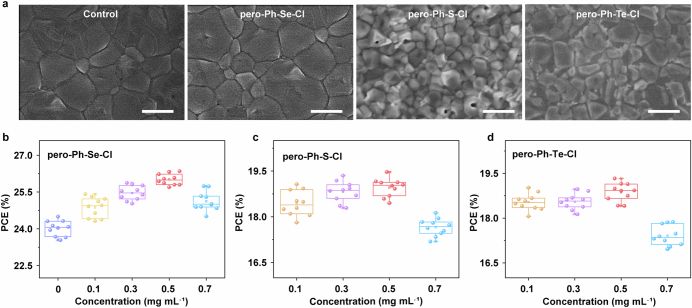
The performance of pero-SCs with different chalcogenides. **a**, SEM images of perovskite films based on different chalcogenides. The scale bar is 1 µm. **b-d**, Photovoltaic parameters for the devices with different concentration of different chalcogenides under AM1.5 G illumination (10 devices for each type). Source Data

To develop efficient coordinative materials, we compared the functional groups of common additives and found that chalcogenide elements contain several lone pair electrons, which could achieve strong coordination ability. Considering the solubility of the material, we introduced the organic functional group benzene (Ph) and synthesized a series of chalcogenides additives (Supplementary Fig. 16), such as benzenesulfenyl chloride (Ph-S-Cl), phenylselenenyl chloride (Ph-Se-Cl) and benzenetellurenyl chloride (Ph-Te-Cl). The major roles of Cl are speculated in Supplementary Note 4. By comparison, the Ph-Se-Cl-based pero-SCs showed the highest PCE (Extended Data Fig. 4 and Supplementary Table 4, as denoted in Supplementary Note 5). Therefore, we explored the ability of Ph-Se-Cl in perovskite lattice strain regulation.

**Fig. 3:**
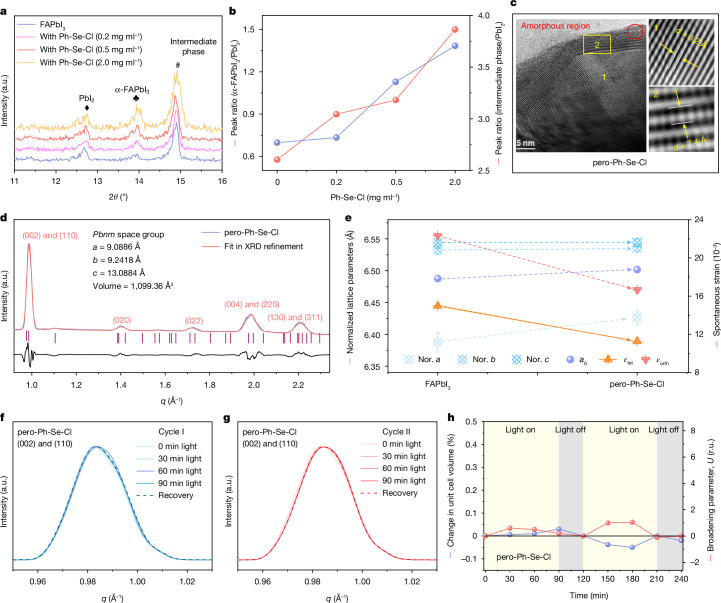
Mitigating cycled lattice strain through Ph-Se-Cl modification. **a**, X-ray diffraction of the wet perovskite films (before annealing) with different amounts of Ph-Se-Cl. **b**, The peak ratio of α-FAPbI_3_/PbI_2_ and intermediate phase/PbI_2_. **c**, High-resolution transmission electron microscopy images of the pero-Ph-Se-Cl film. Parameters *d* represents the interplanar spacing. **d**, Structural refinement (La Bail method) of the integrated GIWAXS profile recorded from the pero-Ph-Se-Cl films. **e**, Normalized (Nor.) lattice parameters and calculated spontaneous strain of FAPbI_3_ and the pero-Ph-Se-Cl samples. Parameters *ε*_tet_ and *ε*_orth_ respectively represent the degenerate tetragonal and orthorhombic symmetry-adapted strains which emerge during the phase transitions. Parameters *a*, *b*, and *c* are the normalized lattice parameters of the perovskite; *a*_0_ is estimated by taking the cube root of the normalized unit cell volume. **f**,**g**, Integrated profiles obtained from in situ GIWAXS maps of the pero-Ph-Se-Cl films (**f**, the first cycle; **g**, the second cycle). **h**, Evolution of the unit cell volume and the peak broadening parameters for the pero-Ph-Se-Cl samples during the in situ GIWAXS measurements. Source Data

We first monitored the effect of the Ph-Se-Cl additive on the crystallization process of perovskites in a two-step method. The mixture of Ph-Se-Cl and formamidinium iodide (FAI) in isopropanol solution shows an absorption spectrum that is similar to that of the Ph-Se-I (Supplementary Fig. 19a), indicating that ion exchange occurs between Ph-Se-Cl and FAI (Ph-Se-Cl + I^−^ → Ph-Se-I + Cl^−^). By tracking the X-ray diffraction of the intermediate state (PbI_2_ react with organic halides without further annealing), we found that the formation of the perovskite phase as well as the intermediate phase are promoted whereas the remaining PbI_2_ phase is reduced with Ph-Se-Cl (Fig. 3a,b). The increased perovskite/PbI_2_ and intermediate/PbI_2_ ratios indicate that the Ph-Se-Cl additive can regulate the crystallization of the perovskite in a two-step method^30,31^.

**Extended Data Fig. 5:**
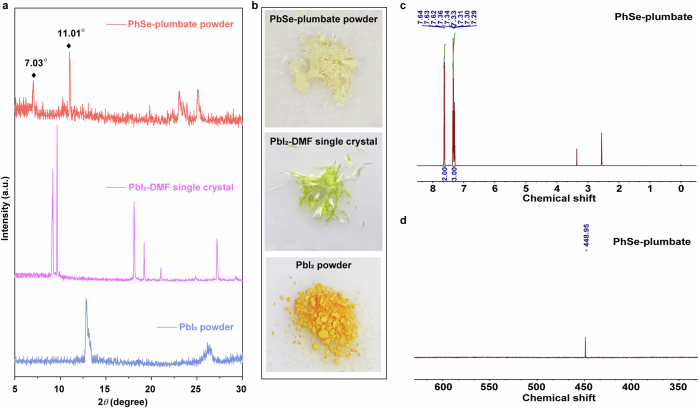
The formation of PhSe-plumbate. **a**, XRD pattern of pure PbI_2_ powder, PbI_2_**-**DMF single crystal and PhSe-plumbate powders. **b**, The corresponding pictures. **c**, ^1^H NMR spectrum of PhSe-plumbate. **d**, ^77^Se NMR spectrum of PhSe-plumbate. Source Data

We further explored the remaining products of Ph-Se-Cl in the final perovskite film. By directly mixing Ph-Se-Cl and PbI_2_, there is a clear C–Se vibration signal shift after Ph-Se-Cl reacting with PbI_2_ in the Fourier transform infrared spectra (Supplementary Fig. 19b). In addition, the shift of Pb 4*f* signals and the appearance of Se 3*d* signals in X-ray photoelectron spectroscopy (Supplementary Fig. 20) also confirm the reaction between Pb^2+^ and Ph-Se-Cl. In our efforts to cultivate single crystals from Ph-Se-Cl and PbI_2_ blends, we only obtained some pale yellowish powders with diffraction peaks at around 7.03° and 11.01° (Extended Data Fig. 5a,b). To further verify the composition of these yellowish powders, we carried out nuclear magnetic resonance measurements and the characteristic peaks of the Ph and Se were detected (Extended Data Fig. 5c,d). Thus, we speculate that the product of Ph-Se-Cl and PbI_2_ is a kind of PhSe-plumbate located at the grain boundaries in the final perovskite film, as observed in the high-resolution transmission electron microscopy and GIWAXS measurements (Fig. 3c and Supplementary Figs. 21 and 22).

**Extended Data Fig. 6:**
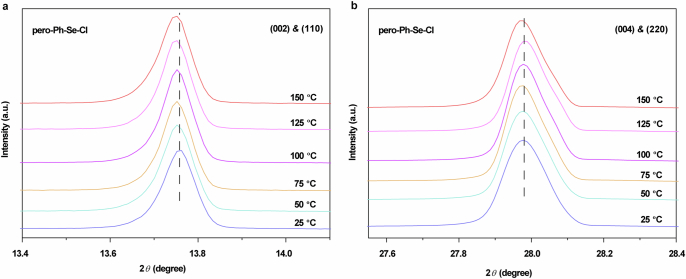
The pseudo-cubic phase for pero-Ph-Se-Cl. Temperature-dependent XRD patterns (cooling process) of the pero-Ph-Se-Cl film. Source Data

After revealing the modification of Ph-Se-Cl on the perovskite film (denoted as pero-Ph-Se-Cl), we moved forward to explore the lattice distortion of pero-Ph-Se-Cl. Unlike the pure FAPbI_3_ film with the orthorhombic phase at room temperature, detailed GIWAXS analysis indicated that the pero-Ph-Se-Cl has a higher symmetric pseudo-cubic phase at room temperature (Fig. 3d and Extended Data Fig. 6), suppressing the lattice distortions. This is probably owing to the crystallization regulation through Ph-Se-Cl^27^. In addition, the characteristic diffraction peaks of the (002)/(110) and (004)/(220) planes remain symmetric in the angle-dependent GIWAXS measurements, revealing a uniform crystallization in the whole pero-Ph-Se-Cl film (Supplementary Fig. 23). The more symmetric lattice also effectively releases the spontaneous strain (Fig. 3e and Supplementary Table 5).

In addition to the spontaneous strain release with a more symmetric lattice at room temperature, we also monitored the lattice strain evolution in the pero-Ph-Se-Cl film during in situ GIWAXS measurements that mimic the cycling mode. The diffraction peaks of the perovskite showed a negligible shift during the whole cycling process, indicating that the addition of Ph-Se-Cl hinders the lattice volume changes (Fig. 3f,g), which is probably linked to the enhanced lattice symmetry and anchoring effects from PhSe-plumbates on grain boundaries, leaving little room for light/thermal-driven expansion. As a result, the unit cell volume and the diffraction peak broadening all show negligible changes during the cycling mode (Fig. 3h and Supplementary Fig. 24). The more symmetric FAPbI_3_ perovskite phase, together with the large steric effect of the PhSe-plumbates at grain boundaries, makes the perovskite lattice less sensitive to illumination and temperature cycling, thus hindering the lattice strain-induced perovskite degradation in the whole cycling mode.

## Stability improvement by Ph-Se-Cl

With the mitigated lattice strain during the cycling mode, the pero-SCs are expected to be free of the detrimental effects caused by plastic deformation, for example, film cracking, deep traps accumulation and ion motion activation. As a result, with lattice strain regulation, the aged pero-Ph-Se-Cl film retains a compact morphology (Supplementary Fig. 25) and the aged device shows one magnitude lower tDOS than the control device in trap band II after ageing for 14 day/night cycles (Supplementary Fig. 26). In addition, the *E*_a_ of ion motion under illumination increases from about 0.184 eV to about 0.381 eV in the continuous mode and from about 0.131 eV to about 0.377 eV in the cycling mode (Supplementary Figs. 27 and 28). The much-enhanced *E*_a_ minimized the ion migration from the active layer to the HTL (Supplementary Figs. 29 and 30).

**Fig. 4:**
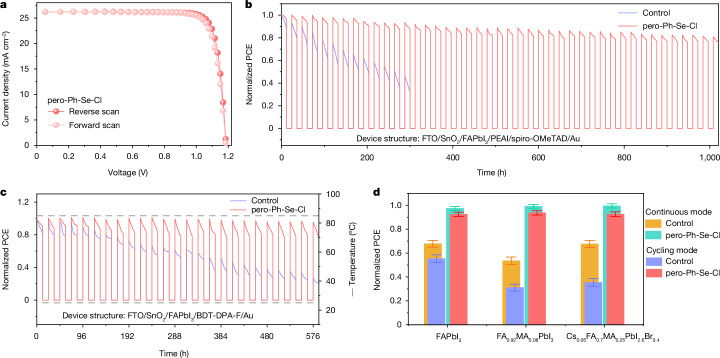
Stability of the pero-Ph-Se-Cl-based devices. **a**, Current–voltage curves of the pero-SCs. **b**, Stability of the pero-SCs based on FAPbI_3_/PEAI/spiro-OMeTAD in the cycling mode. **c**, Stability of the pero-SCs based on FAPbI_3_/BDT-DPA-F in the cycling working. The temperature of the device fluctuated from about 85 °C under illumination to room temperature in the dark. **d**, Stability statistical graphs of the pero-SCs based on FAPbI_3_, FA_0.92_MA_0.08_PbI_3_ and Cs_0.05_FA_0.7_MA_0.25_PbI_2.6_Br_0.4_ working in the continuous mode (108 h) and the cycling mode (9 day/night cycles, illumination 108 h). Error bars represent the standard deviation of 10 devices for each condition. Source Data

The lower density of deep traps and the higher *E*_a_ of ion motion can effectively slow down the degradation rate of pero-SCs. In the continuous mode (Supplementary Fig. 31), the pero-Ph-Se-Cl-based device (certified PCE of 26.3%; Fig. 4a and Supplementary Fig. 32) maintains over 90% of its initial efficiency after 1,000 h, whereas the control device (maximum PCE of 24.5%; Supplementary Table 6) retains only about 44% of its initial PCE after 375 h. More importantly, the stability of the pero-Ph-Se-Cl-based pero-SCs in the cycling mode is significantly improved (Fig. 4b). The control device has only about 39% of its initial PCE after ageing for 13 day/night cycles, whereas the pero-Ph-Se-Cl-based device maintains over 80% of its initial PCE after ageing for 43 day/night cycles (one of the best stability results among unencapsulated spiro-OMeTAD-based pero-SCs).

**Extended Data Fig. 7:**
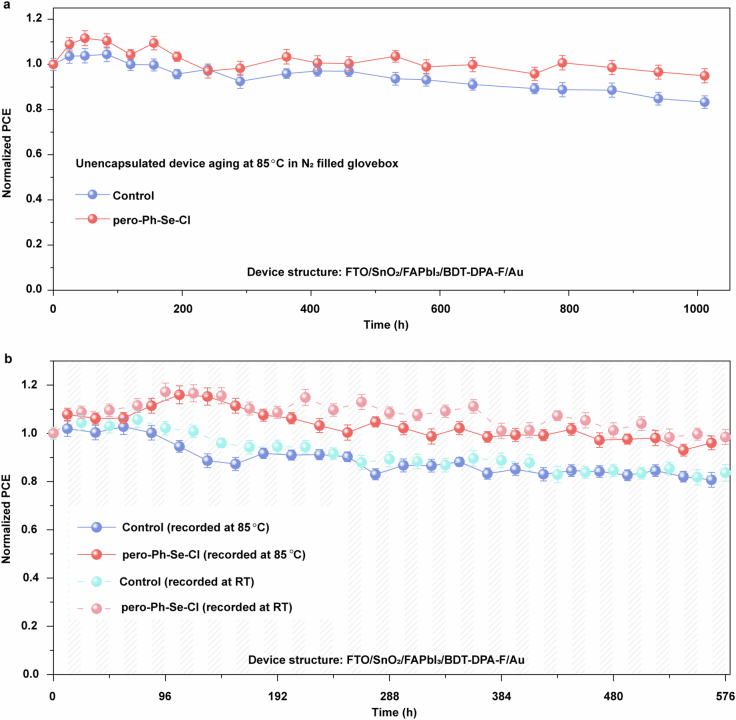
Thermal stability of the pero-Ph-Se-Cl-based devices. (Device structure: FTO/SnO_2_/FAPbI_3_/BDT-DPA-F/Au). **a**, PCE tracking of the pero-SCs without and with Ph-Se-Cl modification under 85 °C in a N_2_-filled glovebox. Data from six cells are collected and presented as mean values  ±  standard error of the mean. **b**, PCE tracking of the pero-SCs without and with Ph-Se-Cl modification under the cycled thermal (12 h RT and 12 h 85 °C) working modes according to the ISOS-T-1 suggested protocol. Source Data

To further explore the stability improvement from lattice strain regulation using Ph-Se-Cl under relatively harsh conditions, we used a more stable 7,7′-(4,8-bis(5-(2-ethylhexyl)-4-fluorothiophen-2-yl)benzo[1,2-b:4,5-b′]dithiophene-2,6-diyl)bis(N,N-bis(4-methoxyphenyl)benzo[c][1,2,5]thiadiazol-4-amine) (BDT-DPA-F) HTL to replace spiro-OMeTAD^32^. We carried out the cycling mode with a temperature range of room temperature to about 85 °C. As shown in Fig. 4c, the pero-Ph-Se-Cl-based device retains up to about 96%, whereas the control device retains only about 27% of its initial efficiency after 25 day/night cycles. We also performed stability measurements under other ISOS protocols. In the continuous thermal ageing measurements (according to ISOS-D-1 protocol; Extended Data Fig. 7a), the pero-Ph-Se-Cl-based devices showed a very small PCE decay (average of about 5%) after 1,000 h, whereas the PCE of the control devices decreases by nearly 20% during the same period. In the cycled thermal ageing measurements (according to ISOS-T-1 protocol; Extended Data Fig. 7b), the pero-Ph-Se-Cl-based device retains up to about 96% at 85 °C and about 98% at room temperature, whereas the control device retains about 80% at 85 °C and about 84% at room temperature of its initial efficiency after 25 thermal cycles. These results under varied ISOS stability standard tests demonstrate that the lattice strain regulation strategy using Ph-Se-Cl additive can significantly stabilize the perovskite lattice and improve the stability of the pero-SCs.

**Extended Data Fig. 8:**
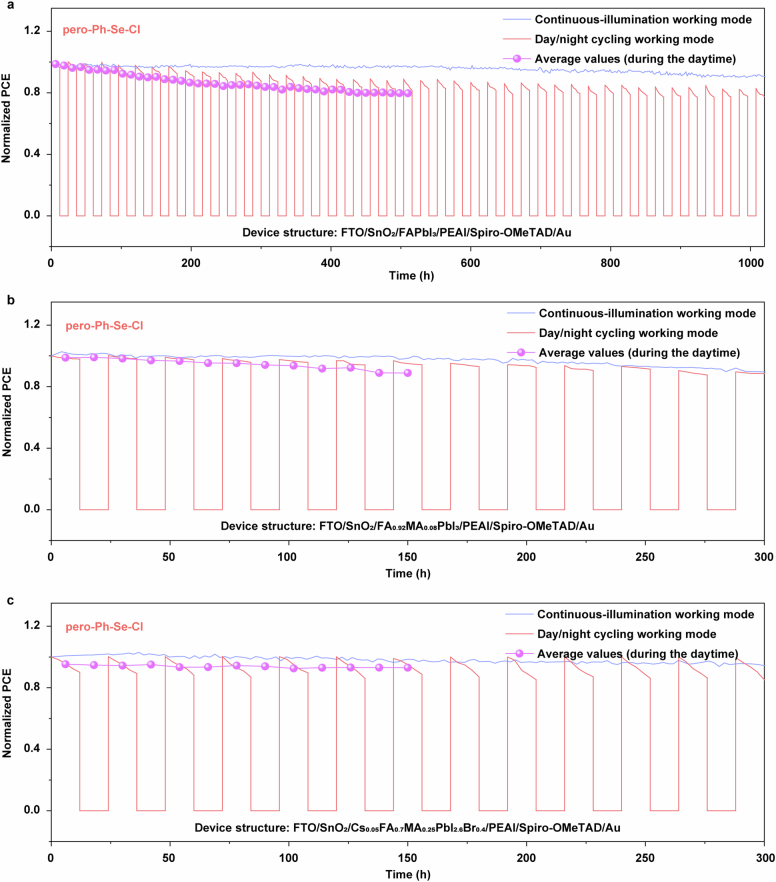
The narrow PCE-degradation gap between the two working modes. **a-c**, PCE tracking of the pero-SCs with Ph-Se-Cl modification based on **a**, FAPbI_3_, **b**, FA_0.92_MA_0.08_PbI_3_ and **c**, Cs_0.05_FA_0.7_MA_0.25_PbI_2.6_Br_0.4_ three different active layer compositions under the continuous-illumination and day/night cycling working modes. Source Data

The degradation rates of the pero-Ph-Se-Cl-based device in the continuous and cycling modes are also comparable to each other after effective lattice strain regulation; about 96% and about 90% of the initial efficiency are maintained after 156 h in the continuous mode and the cycling mode with the same illumination time, respectively (Fig. 4d). In contrast, under the same working conditions, the control device maintains only about 58% and about 39% of the initial PCE, respectively. The devices based on other perovskite active layers with the Ph-Se-Cl modification, such as FA_0.92_MA_0.08_PbI_3_ and Cs_0.05_FA_0.7_MA_0.25_PbI_2.6_Br_0.4_, also show greatly enhanced day/night cycling stability and similar PCE decay dynamics between the cycling mode and the continuous mode (Extended Data Fig. 8 and Supplementary Figs. 31 and 33). The comparable lifetime under the cycling and continuous modes after Ph-Se-Cl modification shows that lattice strain regulation by the Ph-Se-Cl additive effectively mitigates the extrinsic degradation factors induced by the cycling mode, which is promising to pave the way for commercialization of pero-SCs. Some other additives, such as butylammonium chloride, which could assist with more ordered perovskite crystallization, and β-poly(1,1-difluoroethylene), which could regulate the lattice strain through polymer anchoring^15,33^, could also effectively improve the lifetime of pero-SCs in the cycling mode (Supplementary Fig. 34), indicating the generality of lattice strain regulation in minimizing the difference in lifetime extracted from the continuous and cycling modes.

## Methods

### Materials

Fluorine-doped tin oxide (FTO) glass was purchased from South China Xiang Science and Technology. The SnO_2_ colloid precursor (tin (IV) oxide, 15% in water (H_2_O) colloidal dispersion) was purchased from Alfa Aesar. The SnCl_2_·2H_2_O, 4-tert-butylpyridine (tBP), chlorobenzene, isopropanol (IPA), lead bromide (PbBr_2_), dimethylformamide (DMF) and dimethyl sulfoxide (DMSO) were purchased from Sigma Aldrich. Lead iodide (PbI_2_) was purchased from Alfa Aesar. Caesium iodide (CsI), caesium chloride (CsCl), formamidinium iodide (FAI), methylammonium bromide (MABr), methylammonium iodide (MAI), methylammonium chloride (MACl), phenethylammonium iodide (PEAI), 2,2,7′,7′-tetrakis(*N*,*N*-di(4-methoxyphenyl)amine)-9,9′-spirobifluorene (spiro-OMeTAD) and bis(trifluoromethane)sulfonimide lithium salt (LiTFSI) were all purchased from Xi’an Polymer Light Technology. The diphenyl disulfide (99%) and sulfonyl chloride (98%) were purchased from Aladdin. The diphenyl diselenide (98.5%) was purchased from JK and the diphenyl ditelluride (98%) was purchased from Rhawn.

### Material synthesis

#### Synthesis of Ph-Se-Cl

Sulfonyl chloride (0.67 ml, 5 mmol) was added dropwise to a 50-ml round-bottomed flask saturated with argon, containing a solution of diphenyl diselenide (1.56 ml, 5 mmol) in anhydrous dichloromethane (20 ml) at 0 °C, and the solution was stirred. Then the solution was further stirred for 1 h at room temperature. Finally, the obtained mixture was concentrated under reduced pressure to give an orange solid, which was used without further purification. ^1^H NMR (400 MHz, CDCl_3_): *δ* 7.82–7.79 (m, 2H), 7.43–7.41 (m, 3H). ^13^C NMR: (101 MHz, CDCl_3_) *δ* 134.4, 131.7, 130.6, 129.6.

#### Synthesis of Ph-S-Cl

Sulfonyl chloride (0.67 ml, 5 mmol) was added dropwise to a 50-ml round-bottomed flask saturated with argon, containing a solution of diphenyl disulfide (1.09 ml, 5 mmol) in anhydrous dichloromethane (20 ml) at 0 °C, and the solution was stirred. Then the solution was further stirred for 1 h at room temperature. Finally, the obtained mixture was concentrated under reduced pressure to give a dark red oil, which was used without further purification. ^1^H NMR (400 MHz, CDCl_3_): *δ* 7.67–7.63 (m, 2H), 7.42–7.38 (m, 3H). ^13^C NMR (101 MHz, CDCl_3_) *δ* 131.8, 130.1, 129.4, 127.6.

#### Synthesis of Ph-Te-Cl

Sulfonyl chloride (0.67 ml, 5 mmol) was added dropwise to a 50-ml round-bottomed flask saturated with argon, containing a solution of diphenyl ditelluride (2.05 ml, 5 mmol) in anhydrous dichloromethane (20 ml) at 0 °C, and the solution was stirred. Then the solution was further stirred for 1 h at room temperature. Finally, the obtained mixture was concentrated under reduced pressure to give a black solid, which was used without further purification. ^1^H NMR (400 MHz, CDCl_3_): *δ* 8.10–8.07 (m, 2H), 7.55–7.52 (m, 3H). ^13^C NMR: (101 MHz, CDCl_3_) *δ* 135.5, 133.8, 131.8, 130.1.

### Precursor preparation

For FAPbI_3_ precursor solution, 1.5 M PbI_2_ powder was dissolved in 1 ml DMF and DMSO (9:1, volume/volume), with stirring at 70 °C for 6 h in a nitrogen-filled glovebox. FAI 90 mg and MACl 15 mg were dissolved in 1 ml IPA solution and stirred at room temperature in a nitrogen-filled glovebox until fully dissolved.

For FA_0.92_MA_0.08_PbI_3_ precursor solution, 1.5 M PbI_2_ powder was dissolved in 1 ml DMF and DMSO (9:1, volume/volume), with stirring at 70 °C for 6 h in a nitrogen-filled glovebox. FAI 90 mg, MAI 6.39 mg and MACl 9 mg were dissolved in 1 ml IPA solution and stirred at room temperature in a nitrogen-filled glovebox until fully dissolved.

For Cs_0.05_FA_0.7_MA_0.25_PbI_2.6_Br_0.4_ precursor solution, 1.37 M PbI_2_, 0.20 M PbBr_2_, 1.29 M FAI, 0.20 M MABr and 0.40 M MACl were dissolved in 1 ml DMF and DMSO (4:1, volume/volume). Then 44 μl CsI-DMSO precursor (1.5 M) was added in 1 ml perovskite precursor. The precursor solution was stirred for 5 h and was filtered before use.

### Device fabrication

FTO-coated glass substrates were rinsed with deionized water, acetone and isopropyl alcohol by ultrasonication, sequentially, and then dried with nitrogen. Then the substrate was spin-coated with a thin layer of nanoparticle-type SnO_2_ (NP-SnO_2_) (22.6 mg SnCl_2_·2H_2_O dissolved in 1 ml ethanol) at 4,000 rpm for 30 s, and annealed in ambient air at 150 °C for 30 min. The prepared NP-SnO_2_ film was treated with ultraviolet ozone for 10 min to improve the penetration/contact of the colloid SnO_2_ (Col-SnO_2_) solution (the SnO_2_ colloid precursor was diluted to 28 mg ml^−1^ with deionized water). The intermeshing SnO_2_ (Im-SnO_2_) films were obtained by spin-coating Col-SnO_2_ precursor solution on the ultraviolet-ozone-treated NP-SnO_2_ films at 6,000 rpm for 30 s, followed by thermal annealing in ambient atmosphere.

For the FAPbI_3_ layer deposition, 1.5 M PbI_2_ was spin-coated onto SnO_2_ at 1,500 rpm for 30 s, and annealed at 70 °C for 1 min. Then ammonium salt solution was dynamically spun onto the PbI_2_ film at 1,800 rpm for 30 s, followed by thermal annealing at 150 °C for 15 min in ambient air conditions (about 40% humidity). For the pero-Ph-Se-Cl film, 0.5 mg ml^−1^ Ph-Se-Cl was dissolved in ammonium salt solution.

For the FA_0.92_MA_0.08_PbI_3_ layer deposition, 1.5 M PbI_2_ was spin-coated onto SnO_2_ at 1,500 rpm for 30 s, and annealed at 70 °C for 1 min. Then ammonium salt solution was dynamically spun onto the PbI_2_ film at 2,300 rpm for 30 s, followed by thermal annealing at 150 °C for 15 min in ambient air conditions (about 25% humidity). For the pero-Ph-Se-Cl film, 0.5 mg ml^−1^ Ph-Se-Cl was dissolved in ammonium salt solution.

For Cs_0.05_FA_0.7_MA_0.25_PbI_2.6_Br_0.4_, the perovskite precursor solution was spin-coated at 1,000 rpm for 10 s and subsequently at 5,000 rpm for 30 s; 120 µl chlorobenzene as the anti-solvent was poured onto the spinning substrate at 15 s in the second spinning step. For the pero-Ph-Se-Cl film, 0.5 mg ml^−1^ Ph-Se-Cl was dissolved in chlorobenzene. The perovskite films were annealed at 105 °C for 30 min in dry air (about 20% relative humidity).

Then 5 mg ml^−1^ PEAI solution in IPA was spin-coated onto the perovskite surface at 5,000 rpm for the surface passivation. Later, the HTL was deposited on top of the perovskite layer at a spin rate of 4,000 rpm for 30 s using spiro-OMeTAD solution, which consisted of 72.3 mg spiro-OMeTAD), 35 μl LiTFSI stock solution (260 mg LiTFSI in 1 ml acetonitrile), 30 μl tBP and 1 ml chlorobenzene. Metal electrodes (80 nm Au) were deposited on the HTL through a thermal evaporation method under a vacuum degree higher than 3 × 10^−6^ torr to accomplish the solar cell fabrication. A 0.0624-cm^2^ shadow mask was used to define the effective working area of the solar cells.

### Characterizations and measurements

#### Electrical measurements

The current–voltage characteristics of the devices were measured with a computer-controlled Keithley 2450 Source Measure Unit under AM1.5G illumination (100 mW cm^−2^) from an SS-F5-3A solar simulator (Enli Technology) without any preconditioning. The light intensity was calibrated by a standard silicon solar cell (SRC-00178, calibrated by Enli Technology) before testing. The current–voltage curves of the devices were measured in forward scan (from −0.2 V to 1.2 V) mode with a scan step length of 0.02 V and a dwell time of 1 ms for each voltage. The external quantum efficiency (EQE) spectra were obtained using a QE-R3011 solar cell spectral response measurement system (Enli Technology). The light intensity at each wavelength was also calibrated with a standard silicon solar cell (RCS103011-E, calibrated by Enli Technology). The aperture areal of the 0.0624-cm^2^ mask was used for testing devices. The activation energy of ion migration, thermal admittance spectroscopy and drive-level capacitance profiling measurements were performed using an Keithley 4200 Semiconductor Characterization System (as mentioned in Supplementary Note 3).

#### Characterizations on perovskite films

The transmittance and absorption spectra were measured with an ultraviolet spectrometer (Agilent Technologies Cary 5000 UV-vis-NIR). The scanning electron microscopy images were collected on an SU8010 produced by Hitachi, where the electron beam was accelerated at 5 kV. X-ray diffraction patterns were collected using X’Pert Pro MPD (PANalytical). High-resolution transmission electron microscopy was performed on a Thermo Fisher Tecnai F20 transmission electron microscope S4 operated at 200 kV. We dissolved the synthesized PhSe-plumbate powder in DMF, ultrasonicated it for 60 s and then transferred it to the copper grid with a pipette. For the theoretical calculations, first-principles calculations were performed under the framework of density functional theory as implemented in the VASP code.

#### Grazing-incidence wide-angle X-ray scattering

GIWAXS measurements were recorded at the Shanghai Synchrotron Radiation Facility. For synchrotron GIWAXS measurements, all the samples were prepared with perovskite solutions on silicon substrates as described in ‘Device fabrication’. The samples were placed on a programmable temperature control stage (temperature set at 55 °C) inside a nitrogen-filled box. To conduct in situ GIWAXS measurements under light, we installed an AM1.5G solar simulator on top of the nitrogen-filled box, where the light comes through the glass window above the sample for continuous illumination while collecting the data. Two-dimensional synchrotron radiation GIWAXS was performed at the BL14B beamline, Shanghai Synchrotron Radiation Facility with a wavelength of 1.23980 Å. The grazing-incidence angle was fixed at 0.5° and the exposure time was set to 40 s for every 30 min interval.

#### Time-of-flight secondary ion mass spectrometry

Time-of-flight secondary ion mass spectrometry measurements were performed on a TOF-SIMS.5 instrument from IONTOF under an analysis chamber pressure of below 1.1 × 10^−9^ mbar at the Instrument Analysis Centre, Shanghai Jiaotong University. Organic imaging with delay extraction mode with a pulsed 30 keV Bi^3+^ (about 0.16–0.28 pA pulsed current) ion beam was applied for high-lateral-resolution mapping (<800 nm) analysis, and the typical analysis area was 100 × 100 μm^2^, with 1 keV Cs^+^ ion beam sputtering at the same time (about 69.27–82.74 nA current, 300 × 300 μm^2^ sputter raster).

#### Operational stability measurements

Operational stability measurements of the pero-SCs were conducted by a commercial multichannel stability test system (Keithley 2400 source meter) operating in the maximum power point (MPP) tracking mode with a stimulation intensity of 100 mW cm^−2^ (spectra region 410–850 nm, Suzhou D&R Instruments PVLT-G8001M-32B).

## Online content

Any methods, additional references, Nature Portfolio reporting summaries, source data, extended data, supplementary information, acknowledgements, peer review information; details of author contributions and competing interests; and statements of data and code availability are available at 10.1038/s41586-024-08161-x.

## Supplementary information

Supplementary information This file contains Supplementary Notes 1–5, Figs. 1–34, Tables 1–6 and References.

## Source data

Source Data Fig. 1

Source Data Fig. 2

Source Data Fig. 3

Source Data Fig. 4

Source Data Extended Data Fig. 1

Source Data Extended Data Fig. 2

Source Data Extended Data Fig. 3

Source Data Extended Data Fig. 4

Source Data Extended Data Fig. 5

Source Data Extended Data Fig. 6

Source Data Extended Data Fig. 7

Source Data Extended Data Fig. 8

## Figures and Tables

**Extended Data Table 1:**
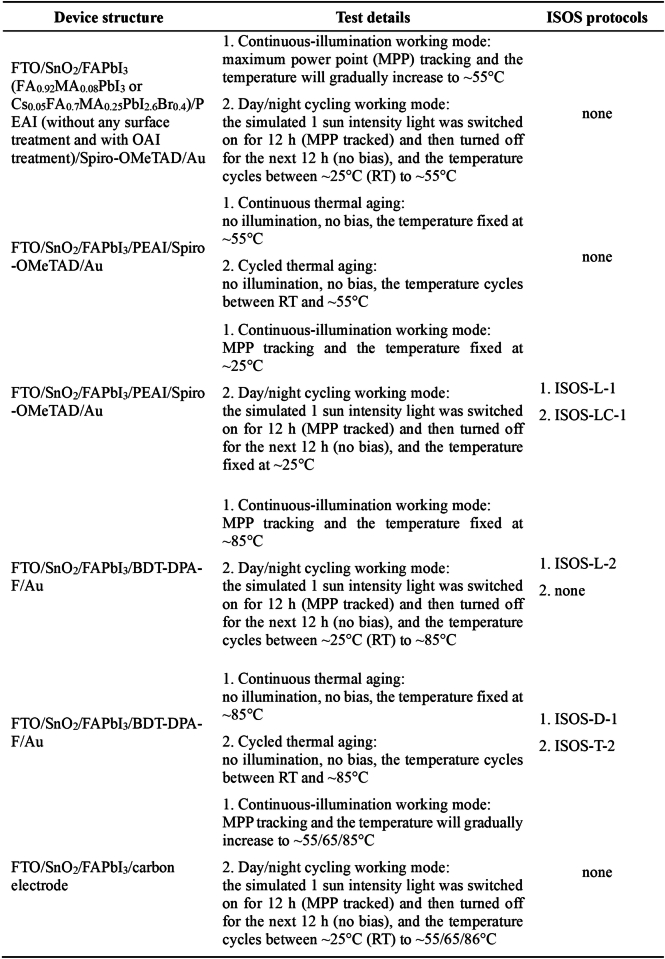
The summary of all ageing conditions used in the study

## Data Availability

The authors declare that the experimental data that support the findings of this paper are available within the article and its Supplementary Information files. Other findings in this study are available from the corresponding authors on reasonable request. Source data are provided with this paper.
