# Supplementary material for: Strain regulation retards natural operation decay of perovskite solar cells
Source: Nature. 2024 Oct 14;635(8040):882–9. doi: 10.1038/s41586-024-08161-x (PMC11602722; doi:10.1038/s41586-024-08161-x)
Supplement: Supplementary file 1 — This file contains Supplementary Notes 1–5, Figs. 1–34, Tables 1–6 and References. [file 41586_2024_8161_MOESM1_ESM.pdf]

nature portfolio

https://doi.org/10.1038/s41586-024-08161-x

Supplementary information

Strain regulation retards natural operation decay of perovskite solar cells

In the format provided by the authors and unedited

Supplementary Materials for

Strain Regulation Retards Natural Operation Decay of Perovskite Solar Cells

Yunxiu Shen[1†], Tiankai Zhang[2†], Guiying Xu[1†], Julian A. Steele[3], Xiankai Chen[4], Weijie Chen[1], Guanhaojie Zheng[5], Jiajia Li[6], Boyu Guo[7], Heyi Yang[1], Yeyong Wu[1], Xia Lin[6], Thamraa Alshahrani[8], Wanjian Yin[9], Jian Zhu[6], Feng Wang[2], Aram Amassian[7], Xingyu Gao[5], Xiaohong Zhang[4,10*], Feng Gao[2*], Yaowen Li[1,6,10*], and Yongfang Li[1,10,11]

[1] Laboratory of Advanced Optoelectronic Materials, Suzhou Key Laboratory of Novel Semiconductor-optoelectronics Materials and Devices, College of Chemistry, Chemical Engineering and Materials Science, Soochow University, Suzhou 215123, China

[2] Department of Physics, Chemistry and Biology (IFM), Linköping University, Linköping 58183, Sweden

[3] Australian Institute for Bioengineering and Nanotechnology and School of Mathematics and Physics, The University of Queensland, Brisbane, QLD 4072, Australia.

[4] Institute of Functional Nano & Soft Materials (FUNSOM), Soochow University, Suzhou, Jiangsu 215123, P. R. China

[5] Shanghai Synchrotron Radiation Facility (SSRF), Zhangjiang Lab, Shanghai Advanced Research Institute, Chinese Academy of Sciences, Shanghai 201204, China

[6] State and Local Joint Engineering Laboratory for Novel Functional Polymeric Materials, Jiangsu Key Laboratory of Advanced Functional Polymer Design and Application, College of Chemistry, Chemical Engineering and Materials Science, Soochow University, Suzhou 215123, China

[7] Department of Materials Science and Engineering and Organic and Carbon Electronics Laboratories (ORaCEL), North Carolina State University, Raleigh, NC 27695, USA

[8] Department of Physics, College of Science, Princess Nourah bint Abdulrahman University, Riyadh 11671, Saudi Arabia

[9] College of Energy, Soochow Institute for Energy and Materials InnovationS (SIEMIS), Soochow University, Suzhou 215006, China

[10] Jiangsu Key Laboratory of Advanced Negative Carbon Technologies, Soochow University, Suzhou 215123, China

[11] Beijing National Laboratory for Molecular Sciences; CAS Key Laboratory of Organic Solids, Institute of Chemistry, Chinese Academy of Sciences, Beijing 100190, China

*Correspondence author. Email: xiaohong_zhang@suda.edu.cn (X.H.Z); feng.gao@liu.se (F.G.); ywli@suda.edu.cn (Y.W.L.)

†These authors contributed equally to this work.

Contents

Supplementary Notes 4
Supplementary Note 1 4
Supplementary Note 2 4
Supplementary Note 3 5
Supplementary Note 4 6
Supplementary Note 5 7
Supplementary Figures 8
Supplementary Fig. 1 8
Supplementary Fig. 2 9
Supplementary Fig. 3 10
Supplementary Fig. 4 11
Supplementary Fig. 5 12
Supplementary Fig. 6 13
Supplementary Fig. 7 14
Supplementary Fig. 8 15
Supplementary Fig. 9 16
Supplementary Fig. 10 17
Supplementary Fig. 11 18
Supplementary Fig. 12 19
Supplementary Fig. 13 20
Supplementary Fig. 14 21
Supplementary Fig. 15 22
Supplementary Fig. 16 23
Supplementary Fig. 17 24
Supplementary Fig. 18 25
Supplementary Fig. 19 26
Supplementary Fig. 20 27
Supplementary Fig. 21 28
Supplementary Fig. 22 29
Supplementary Fig. 23 30
Supplementary Fig. 24 31
Supplementary Fig. 25 32
Supplementary Fig. 26 33
Supplementary Fig. 27 34
Supplementary Fig. 28 35
Supplementary Fig. 29 36
Supplementary Fig. 30 37
Supplementary Fig. 31 38
Supplementary Fig. 32 39
Supplementary Fig. 33 40
Supplementary Fig. 34 41
Supplementary Tables 42

Supplementary Table 1 42
Supplementary Table 2 43
Supplementary Table 3 44
Supplementary Table 4 45
Supplementary Table 5 46
Supplementary Table 6 47
Additional references 48

Supplementary Notes

Supplementary Note 1. The lattice strain analysis of the $FAPbI_3$ film

From the temperature dependent XRD measurements (Extended Data Figs. 3a-b), we found the peak at around 28º is symmetric at 150ºC. When the temperature decreases from 150ºC to RT, this symmetric diffraction peak of (004)/(220) plane becomes asymmetric and its FWHM expands from ~0.11º to ~0.15º (Supplementary Fig. 4a), implying the crystal lattice distortion and symmetry change from annealing temperature to RT.

The stress in the perovskite film mainly originates from the differences in the relative volume change of the perovskite and the underlying substrate after removal from the processing conditions. According to the formula of $\sigma_{\Delta T} = E_p\ (\alpha_S\text{-}\alpha_P)\ \Delta T\ /(1\text{-}\upsilon_P)$, where E_p and υ_P refer to Young's modulus (10 GPa) and Poisson's ratio (0.3) in the perovskite, respectively, while α_s (FTO: 0.85×10^{-5} K^{-1}) and α_p ($FAPbI_3$: 3.5×10^{-5} K^{-1}) represent the linear thermal expansion coefficient of substrate and perovskite, respectively, and ΔT is the temperature gradient during the perovskite fabrication.[1] The calculated stress of the $FAPbI_3$ films is 111.8 Mpa (Supplementary Fig. 4b). Besides, according to the formula, the calculated stress of the $FA_{0.92}MA_{0.08}PbI_3$ and $Cs_{0.05}FA_{0.7}MA_{0.25}PbI_{2.6}Br_{0.4}$ perovskite films are extracted to be 97.5 Mpa and 88.5 Mpa, respectively (Supplementary Fig. 5).

The freestanding perovskite powder was prepared by scraping the as-prepared perovskite films from the substrates (Supplementary Fig. 4c). Compared with the formed perovskite film (at RT) on the FTO/glass substrate, the characteristic peaks of the powder sample shifted to lower diffraction angles, which is a signature for the presence of lattice stress in the film. Note that, regarding the perovskite powder as the stress-free state, the perovskite film suffered from compressive stress to tensile stress during the cooling process (the lattice stress is approximately from 0.3 MPa to -0.2 MPa), and showed a stress-free state at ~70°C (Supplementary Fig. 4d), which corresponds to the lattice expansion caused by light and thermal radiation under the day/night cycling mode.

Supplementary Note 2. Calculation of stress in perovskite film

Perovskite film was deposited on a silicon wafer substrate. Samples are unpacked and stored in the N_2 glovebox, and moved to Linkam Stage for measurement.[2] The laser curvature system consists of two major parts, (i) a 660nm laser source and (ii) a CCD camera. The laser source generates two parallel laser beams through an etalon, with a known interspacing of 3.2 mm. The parallel incident laser beams hit the target substrate with an incident angle of 12.5 degrees, and the reflected laser beams are detected by the CCD camera. The distance L between the laser source/CCD camera and the substrate is calibrated before each measurement, using a flat mirror and a curved mirror with a known radius of curvature of 10 m. The changes in the interspacing of the reflected laser beams (Δd) are tracked using the kSA ICE software. The curvature of the substrate is calculated using equation (S1):

$$K = \frac{\Delta d}{d}\frac{\cos\alpha}{2L} \qquad \text{Eq S1}$$

The stress in the perovskite thin film is evaluated by comparing the substrate curvature before and after film deposition, the magnitude of film stress (σ) is calculated using the Stoney equation (S2):[3]

$$\sigma = \frac{1}{6}\frac{E_s}{1-v_s}\frac{t_s^2}{t_f}\left[\frac{1}{K_f}-\frac{1}{K_s}\right] \qquad \text{Eq S2}$$

E_s is Young's modulus of the substrate, v_s is the Poisson's ratio of the substrate, t_s is the substrate thickness, t_f is the film thickness, K_s is the curvature of the substrate, K_f is the curvature of film and substrate. Silicon substrate with <100> orientation is used in this study, for which E_s = 125 GPa and v_s = 0.302. Therefore, we calculated initial stress values for the samples: $\sigma_{150°C}$ = 88.67 ± 4.62 MPa, $\sigma_{55°C}$ = 73.64 ± 3.38 MPa. The initial values measured using the curvature method appear to be similar to those measured using the temperature-dependent XRD method, and we used $\sigma_{55°C}$ values as the initial stress. Note, all initial stresses are measured at room temperature, $\sigma_{150°C}$ indicates the sample has been heated to 150°C then cooled to RT to measure the initial stress; $\sigma_{55°C}$ indicates the sample has been heated to 55°C then cooled to RT to measure the initial stress.

As shown in Supplementary Fig. 9, when we used the continuous illumination with a fixed temperature at ~25°C, the lattice stress released slowly (releasing ~15 MPa within 200 min). However, when we carried out cycled illumination with a temperature range of ~25°C to ~55°C, the lattice stress showed a rapid reduction (~30 MPa within a few min), indicating that light-induced heat plays a key role in lattice expansion and shrinkage.

Supplementary Note 3. The activation energy of ion migration, TAS and DLCP measurements

The activation energy of ion migration measurement was extracted from the dependence of the conductivity of the $FAPbI_3$ films on temperature. In short, we used a lateral structure device which consists of two Au electrodes deposited on $FAPbI_3$ polycrystalline films, the lateral device structure suppresses the electronic conduction and highlights the ion conducting contribution to the total current. The atom or ion migration rate (r_m) in a solid is determined by the activation energy (E_a) by the Nernst–Einstein relation:

$$\sigma(T) = \frac{\sigma_0}{T}\exp\left(\frac{-E_a}{kT}\right) \qquad \text{Eq S3}$$

Where k is the Boltzmann constant, σ_0 is a constant, and E_a can be derived from the slope of the ln(σ_T)-1/kT relation. During the measurement, a constant electric field of 0.2 V μm^{-1} was applied, which was set to be small to reduce the poling effect.

For the TAS measurement, the DC bias (V) was fixed at 0 V, and the amplitude of the AC bias (δV) was 20 mV. The scanning range of the AC frequency (f) was 0.01−2,000 kHz. The tDOS ($N_T(E_\omega)$) is calculated using the equation:

$$N_T(E_\omega) = -\frac{1}{qkT}\frac{\omega \mathrm{d}C}{\mathrm{d}\omega}\frac{V_{bi}}{W} \qquad \text{Eq S4}$$

where q, k, T, ω and C are elementary charge, Boltzmann's constant, temperature, angular frequency and specific capacitance, respectively. W and V_{bi} are the depletion width and build-in potential, respectively. The demarcation energy:

$$E_\omega = kT\ln(\frac{\omega_0}{\omega}) \quad \text{Eq S5}$$

where ω_0 is the attempt-to-escape angular frequency that equals to $2\pi v_0 T^2$, is derived from the temperature-dependent C–*f* measurements. The reduced attempt-to-escape frequency v_0 is obtained from the fitting of Eq S6 obtained at different *T*.

$$\ln(\frac{T^2}{\omega}) = \frac{E_T}{kT} - \ln(2\pi v_0) \quad \text{Eq S6}$$

In this work, the ω_0 for trap bands were individually derived from the temperature-dependent C–*f* measurements. Each trap band was plotted with a Gaussian distribution in energy and its own ω_0 to determine the trap depth from the band edge. We should note that the TAS method cannot distinguish between states close to the valence band or the conduction band. Temperature-dependent capacitance measurements were carried out in a Lake Shore Cryotronics probe stage with a Lake Shore Cryotronics temperature controller model 336. SMA connected cables and ZN50R d.c./radio frequency probes that were applicable for radio frequency tests up to 1 GHz were equipped with the probe station. Before each measurement, the system was self-calibrated under open-circuit and short-circuit conditions to compensate for any undesired signal from the instrument.

For the DLCP measurements, the *V* was scanning from 0 V to the V_{OC} (for example, 1.2V) for the perovskite solar cells. The DLCP method used a series of variable δV (for example, 20 to 200 mV) to measure the junction capacitance and acquire the capacitance contribution from the trap states by taking advantage of the information embedded in the higher-order terms. The capacitance measured at each δV was recorded and fitted with a polynomial function $C = C_0 + C_1 + \delta V + C_2(\delta V)^2 + \ldots$ to obtain C_0 and C_1. With the determination of C_0 and C_1, the total carrier density (*N*) that includes both free carrier density and trap density at the profiling distance X from the junction barrier is calculated by:

$$N = -\frac{C_0^3}{2q\varepsilon A^2 C_1} \quad \text{Eq S7}$$

where q is the elementary charge, ε is the dielectric constant of the semiconductor (which is 33 for $FAPbI_3$) and *A* is the active area of the junction. The profiling distance from the junction barrier was calculated by $\varepsilon A/C_0$, which was changed by tuning the *V*. For each AC bias, an additional offset DC voltage was applied to keep the maximum forward bias constant. The trap density within a certain trap depth range was calculated by subtracting the total carrier density measured at a larger E_ω (lower AC frequency) with that measured at a smaller E_ω (higher AC frequency).

Supplementary Note 4. Theoretical calculation for the role of Cl on the α-$FAPbI_3$ perovskite structure

First-principles calculations were performed under the framework of density functional theory (DFT) as implemented in the VASP code. The core-valence interactions are treated by the projector augmented wave (PAW) method with a kinetic energy cutoff of 500 eV used for the plane-wave basis. The generalized gradient approximation exchange-correction functional formulated by Perdew, Burke, and Ernzerhof (PBE) is used in all calculations. Single Γ point was used in all calculations for Brillouin zone sampling. The electronic configurations for C,

N, H, Pb, I and Cl elements are $2s^22p^2$, $2s^22p^3$, $1s^1$, $5d^{10}6s^26p^2$, $5s^25p^5$ and $3s^23p^5$. The van der Waals interactions were incorporated into the geometrical optimization process using the DFT-D3 method, with residual forces on atoms converged below 0.01 eV/Å. The self-consistent field calculations were performed using the convergence criterion of 10^{-5} eV/atom.

To further investigate the role of Cl in the perovskite crystal structure, a 3 × 3 × 1 supercell of $FAPbI_3$ was employed and the Cl doping was simulated by individually replacing the I atoms near FA at three distinct sites. As shown in Supplementary Fig. 17a, the lower system energies indicated that Cl can replace I and remain in the perovskite lattice, which most easily emerges in type B. We calculate the formation energy of the α-$FAPbI_3$ using the equation: $\Delta E_f = E_{system} - nE_{FA} - mE_{Pb} - lE_I - tE_{Cl}$, where, E_{system} is the total energy of the perovskite system. E_{FA}, E_{Pb}, E_I, and E_{Cl} are the energies per unit of the reference states, which are the FA, Pb, I and Cl, respectively. *n, m, l,* and *t* are the number of atoms in the system. The above calculation results revealed that the formation energies of the α-$FAPbI_3$ decrease with Cl remaining in lattice, which can ameliorate perovskite crystallization dynamics to result in a more ordered lattice, thus releasing the lattice strain.[4,5] To better understand the role of Cl in the electronic structure, the highest-occupied molecular orbital (HOMO) was calculated and the projected density of states (PDOS) results revealed that Cl has little influence on the DOS of the *p* orbital of I at the HOMO state (Supplementary Figs. 17c-e). In addition, we confirmed that the perovskite unit volume of the Cl-incorporated systems was almost unchanged compared to the control system (Supplementary Fig. 17b). Accordingly, Cl contributes to reducing the formation energy of α-$FAPbI_3$, but has little effect on perovskite HOMO state and lattice volume.

Supplementary Note 5. The study of perovskite films with different chalcogenides

We synthesized a series of chalcogenides additives, such as Ph-S-Cl, Ph-Se-Cl and Ph-Te-Cl, for comparison. The synthesis process and molecular structure characterizations (nuclear magnetic resonance (NMR) spectra) are provided in the Material synthesis part and Supplementary Fig. 16. As for the Ph-S-Cl based perovskite film (0.5 mg mL^{-1}, denoted as pero-Ph-S-Cl), an obvious PbI_2 diffraction peak appeared (Supplementary Fig. 18a), together with weaker absorption compared with that of the pristine 3D perovskite film, as shown by the Ultraviolet-visible spectroscopy (UV–vis) absorption spectra (Supplementary Fig. 18b). It is probably caused by the strong coordination of S atom with Pb^{2+}, which would hinder the crystallization between FAI and PbI_2. As seen from the related SEM images (Extended Data Fig. 4a), the random distributed perovskite grains with small sizes further confirmed the weak crystallization behavior, showing a low PL intensity. When using Ph-Te-Cl additive (0.5 mg mL^{-1}, denoted as pero-Ph-Te-Cl), although no PbI_2 diffraction peaks were observed, the perovskite film showed noncontinuous morphology, lower PL intensity, and weaker UV–vis absorption. Based on this, we hypothesize that the Te atom may have more severe influence on the colloidal formation of perovskite precursors due to the greater electronegativity difference between I and Te (Supplementary Table 3), thus leading to poor crystallization of the perovskite film. In comparison, the Ph-Se-Cl based perovskite film (0.5 mg mL^{-1}, denoted as pero-Ph-Se-Cl) showed compact and uniform crystals with significantly increased grain size, strong PL intensity, and high crystallinity. Therefore, the pero-SCs based on Ph-Se-Cl showed the best performance (Extended Data Figs. 4b-d and Supplementary Table 4).

Supplementary Figures

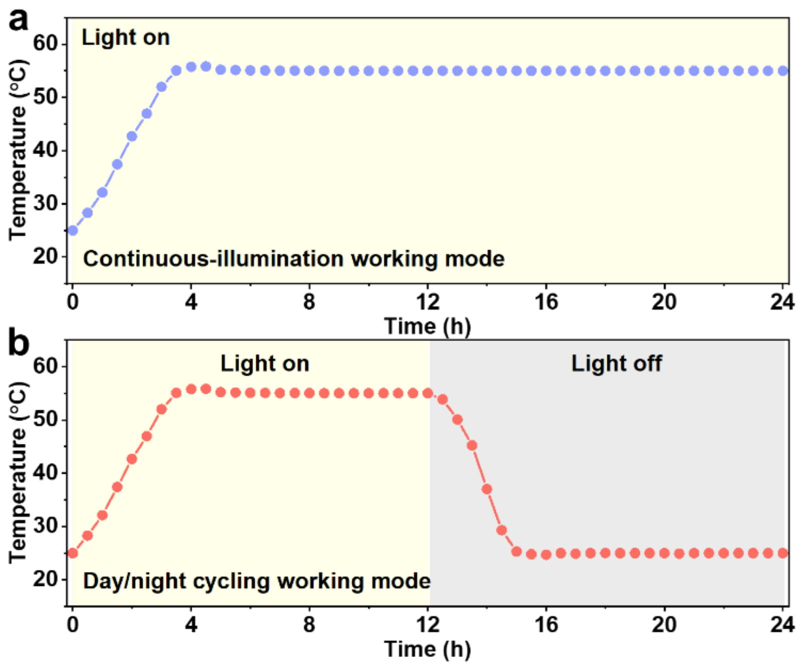

Supplementary Fig. 1 | Faster PCE decay in the day/night cycling working mode. a,b, The temperature fluctuation in the **a,** continuous-illumination mode and **b,** day/night cycling working mode. For the continuous working mode, devices were exposed to 1 sun illumination and tracked at the maximum power point (MPP). For the day/night cycling working mode, the simulated 1 sun intensity light was switched on for 12 h (MPP tracked) and then turned off for the next 12 h (without bias); the temperature of the device fluctuated from ~55°C under illumination to RT in the dark because of the photo-induced radiative heating.

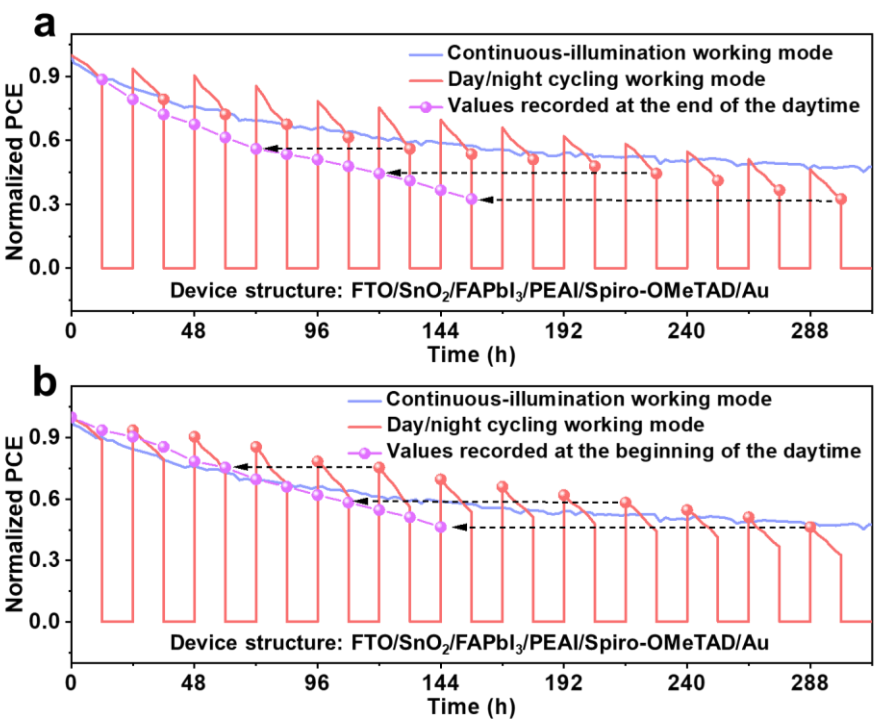

Supplementary Fig. 2 | Faster PCE decay in the day/night cycling working mode. a,b, PCE tracking of the pero-SCs based on $FAPbI_3$ working under the continuous-illumination and day/night cycling modes. (Values recorded at the **a**, end and **b,** beginning of the daytime, respectively). Here, we tested the PCE of the devices every 3 h and recorded five times during 12 h in the day/night cycling mode. The average values of PCE in Fig. 1a is the average value of these five recorded data.

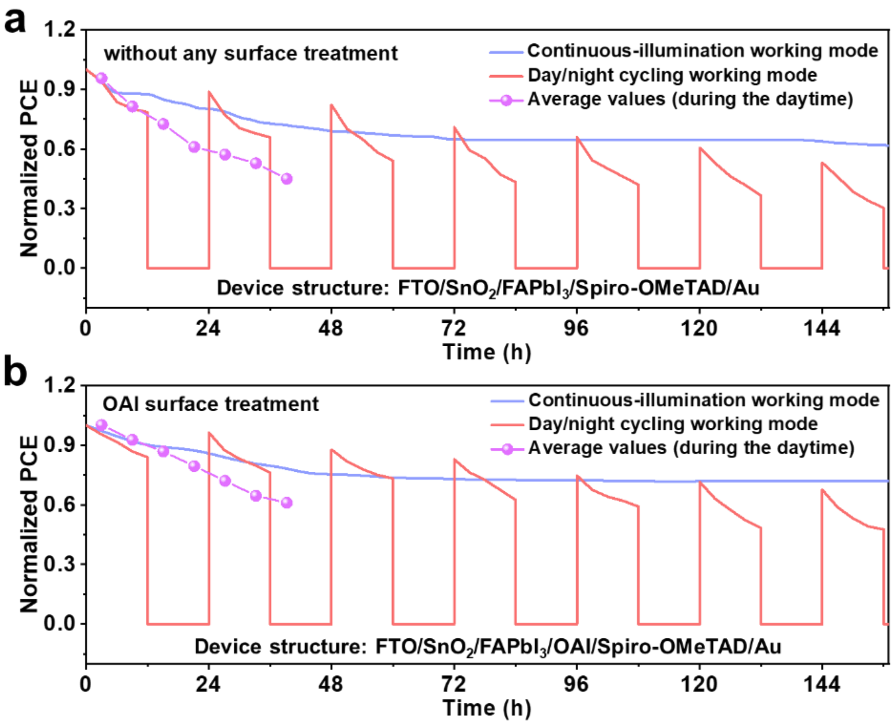

Supplementary Fig. 3 | Faster PCE decay in the day/night cycling working mode. a,b, PCE tracking of the pero-SCs **a,** without any surface passivation and **b,** with OAI passivation working in the continuous-illumination and day/night cycling modes.

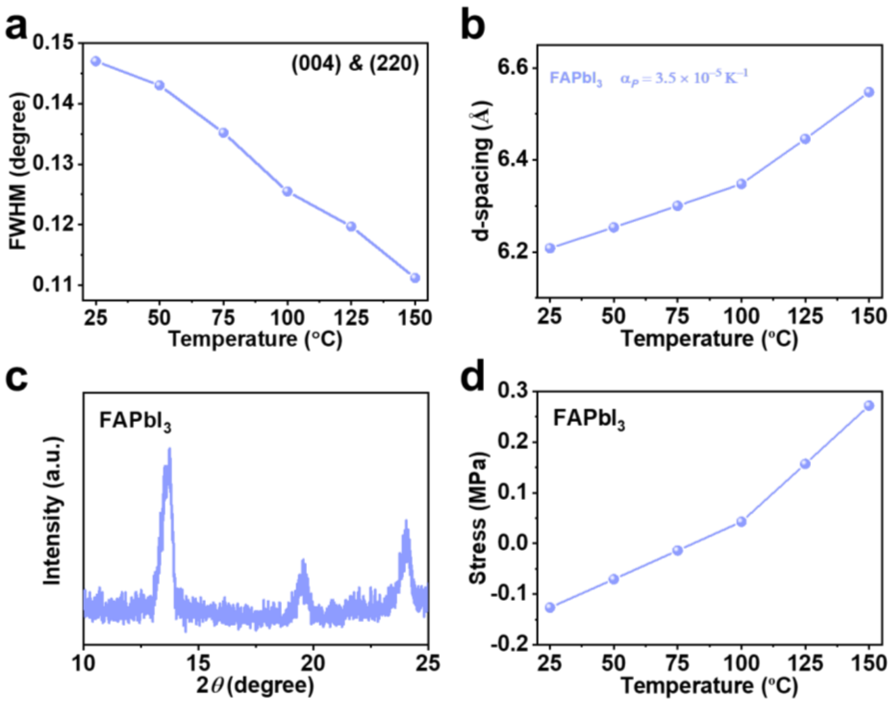

Supplementary Fig. 4 | The lattice strain of $FAPbI_3$. a, Full width at half maximum (FWHM) of the (004)/(220) X-ray diffraction peak as a function of the temperature. **b,** Temperature-dependent *d*-spacing of perovskite films. **c,** XRD pattern of the perovskite powders obtained from the corresponding perovskite films. **d,** Temperature-dependent stress $\sigma_{(100)}$ is calculated by $\sigma_{(100)} = E_p\,(q_{\text{powder}} - q_{(100)})/q_0\,(1-\upsilon_P)$.

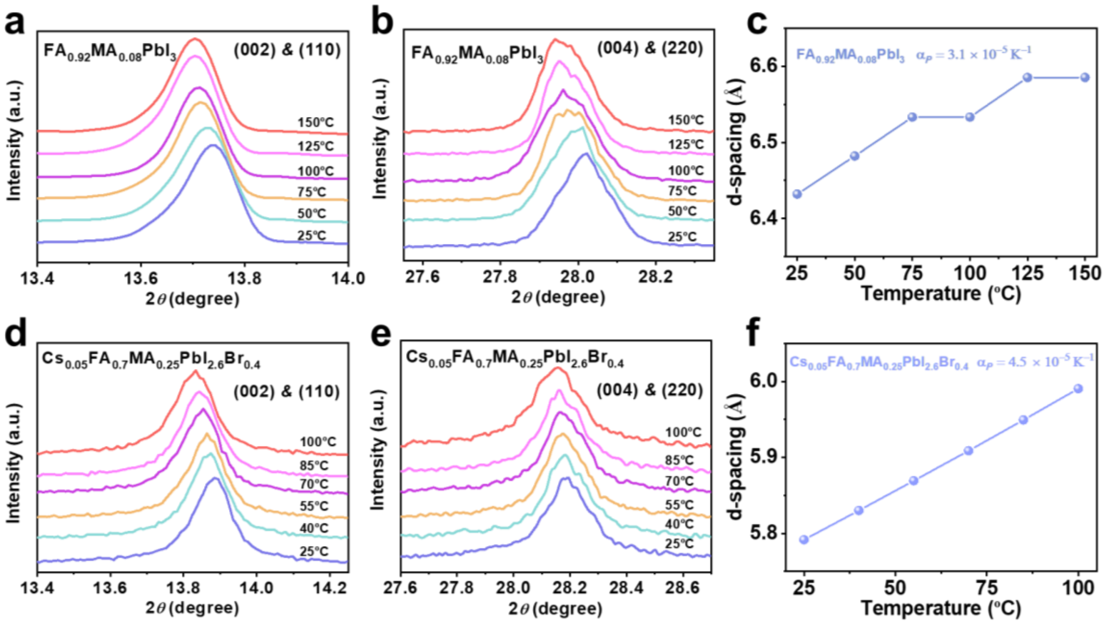

Supplementary Fig. 5 | The lattice strain of different perovskite films. a,b, Temperature-dependent XRD patterns (cooling process) of perovskite films based on $FA_{0.92}MA_{0.08}PbI_3$ (focused on (002)/(110) and (004)/(220) diffractions, respectively). **c,** Temperature-dependent *d*-spacing of $FA_{0.92}MA_{0.08}PbI_3$ film. **d,e,** Temperature-dependent XRD patterns (cooling process) of perovskite films based on $Cs_{0.05}FA_{0.7}MA_{0.25}PbI_{2.6}Br_{0.4}$ (focused on (002)/(110) and (004)/(220) diffractions, respectively). **f,** Temperature-dependent *d*-spacing of $Cs_{0.05}FA_{0.7}MA_{0.25}PbI_{2.6}Br_{0.4}$ film.

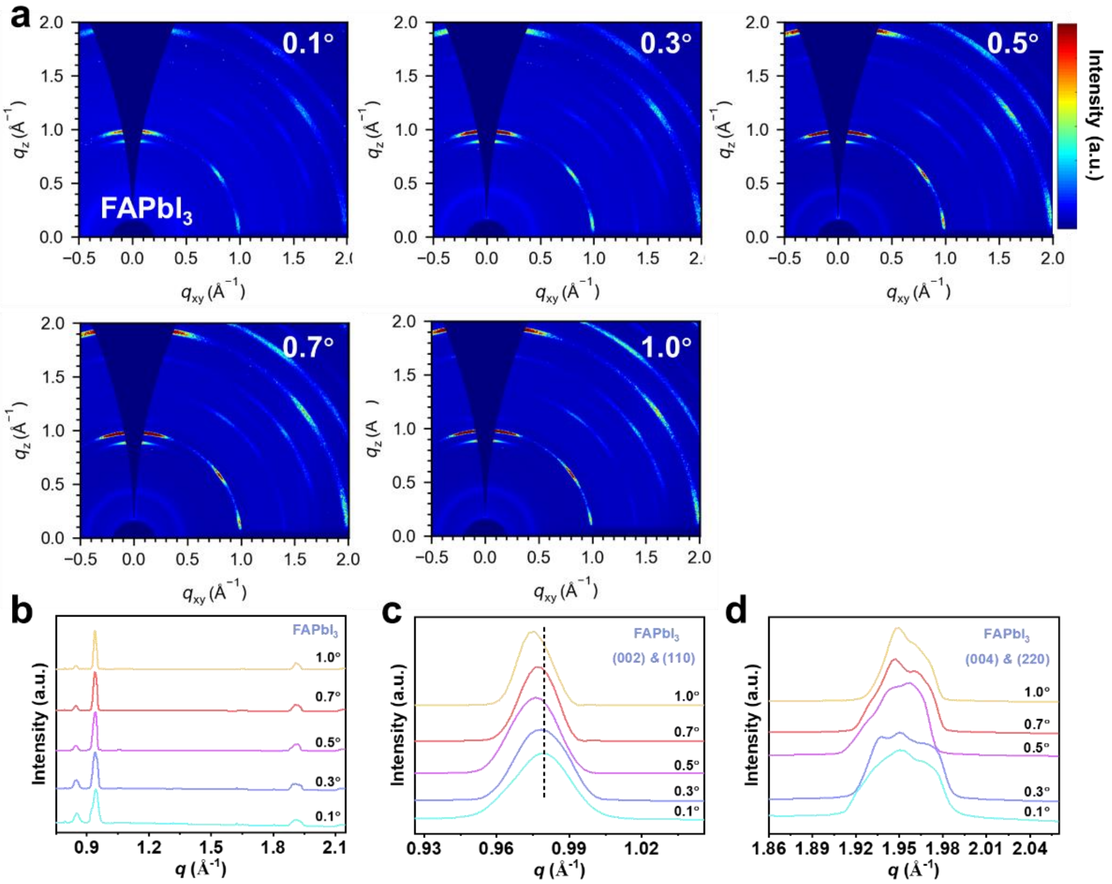

Supplementary Fig. 6 | The orthorhombic phase for $FAPbI_3$. a, The incidence angle dependent GIWAXS maps of the fresh $FAPbI_3$ perovskite film with different incidence angles (0.1°, 0.3°, 0.5°, 0.7° and 1.0°). **b-d,** Integrated profiles obtained from the GIWAXS maps for the $FAPbI_3$ perovskite film with different incidence angles. For the incidence angle below 0.2°, the X-ray source can only penetrate to the top ~10 nm of the perovskite film. For the incidence angle of 1.0°, the X-ray beam would penetrate ~450 nm of the perovskite film.

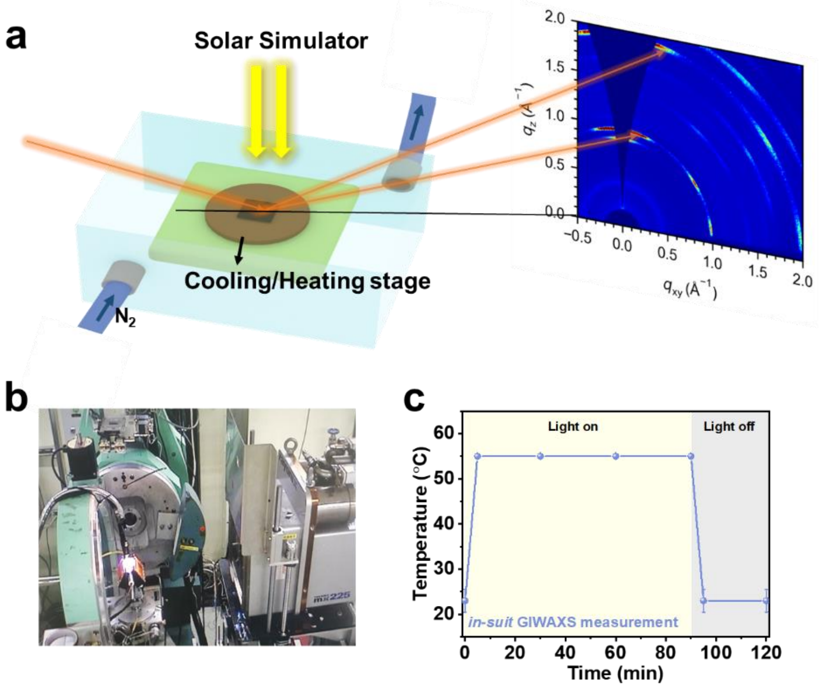

Supplementary Fig. 7 | The measurement conditions for in-situ GIWAXS. a, Schematic picture of the *in-situ* GIWAXS chamber equipped with a cooling/heating plane and a solar simulator working under an N_2 atmosphere. In this case, the cycling started at RT in dark, then the temperature rose to ~55°C within 5 min, and the film was kept under illumination for 90 min under this condition; the film was finally cooled to RT in dark within 30 min. **b,** The picture of the *in-situ* GIWAXS measurement equipment. **c,** The temperature fluctuation in *in-situ* GIWAXS measurements.

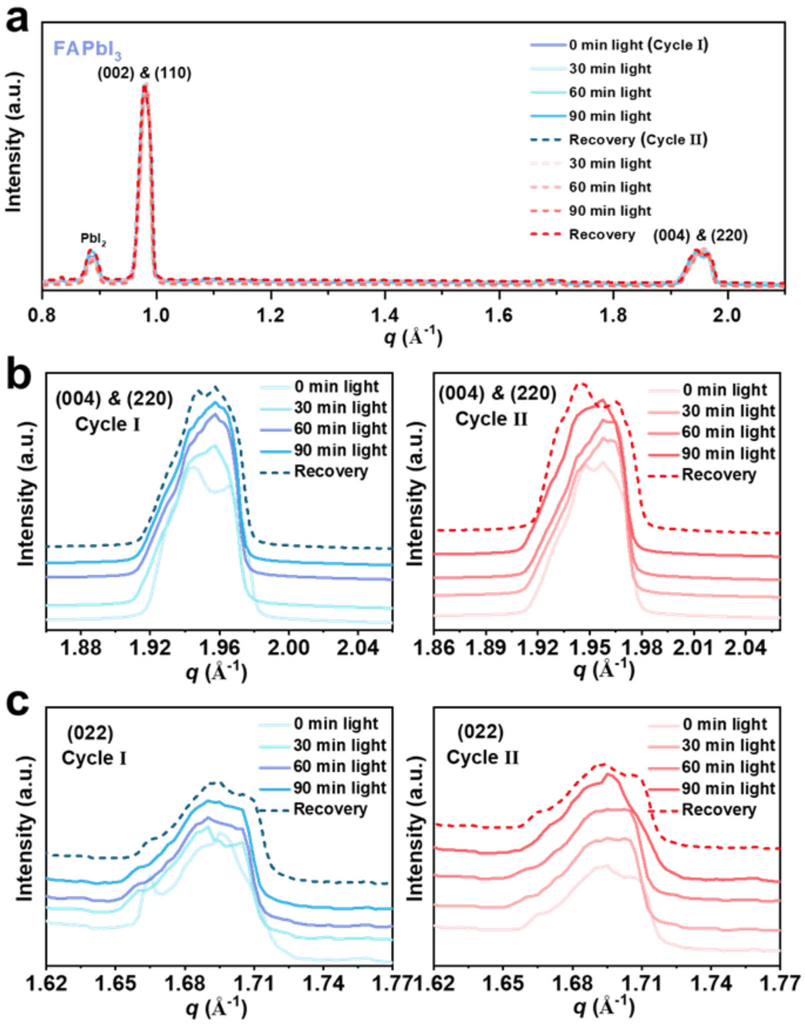

Supplementary Fig. 8 | The crystal cubic-orthorhombic phase transition during the in-situ GIWAXS measurement. a-c, Integrated profiles obtained from the *in-situ* GIWAXS maps for the $FAPbI_3$ perovskite film under illumination from 0 to 90 min, measured at 30-min intervals for the two cycles, and the recovery spectra obtained from the film kept in dark for 30 min (focus on (004)/(200) and (022)).

The shape of the (004)/(200) and (022) peaks changed from asymmetric to symmetric, implying the phase transition from metastable orthorhombic to pseudo-cubic from dark to the daytime period.

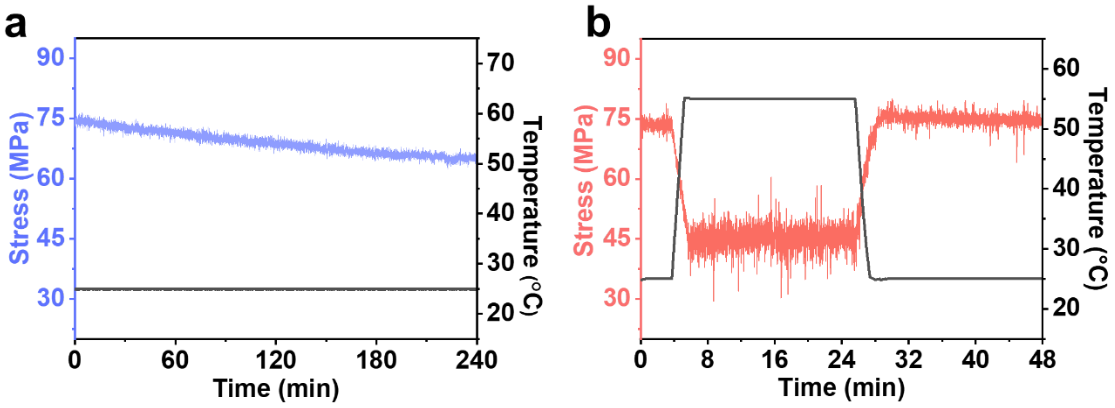

Supplementary Fig. 9 | Film stress calculation extracted from curvature measurements. **a,** Continuous illumination with a fixed temperature at ~25°C. **b,** Cycled illumination with a temperature change between ~25°C to ~55°C.

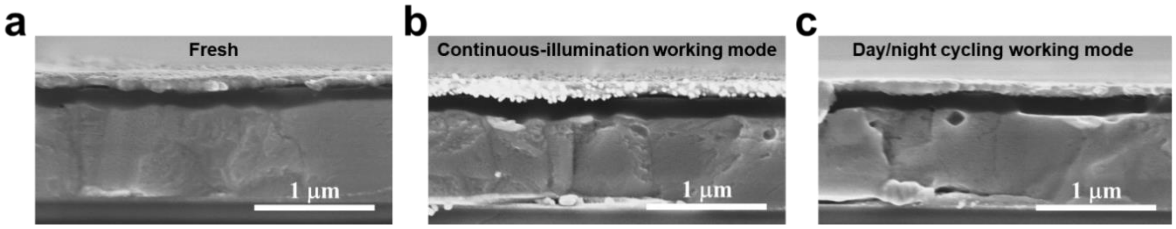

Supplementary Fig. 10 | The degradation of perovskite. a-c, Cross-sectional scanning electron microscopy (SEM) images of pero-SCs based on $FAPbI_3$ before and after aging in the continuous-illumination (156 h) and day/night cycling (13 cycles) working modes.

Some cracks across perovskite and holes between in perovskite/transport layer interfaces in the degraded samples were also observed, indicating a severe plastic deformation for the perovskite under cycling work modes.

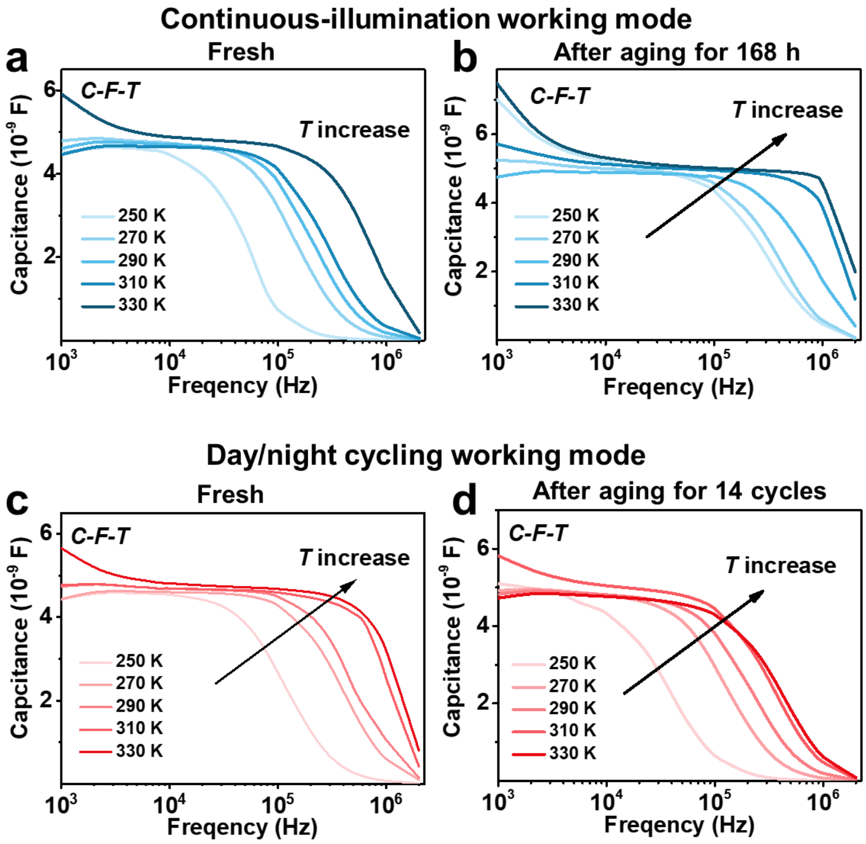

Supplementary Fig. 11 | Defect-evolution dynamics. a,b, Temperature dependent capacitance-frequency spectra of pero-SCs **a,** before and **b,** after aging for 168 h in the continuous-illumination working mode. **c,d,** Temperature dependent capacitance-frequency spectra of pero-SCs **c,** before and after **d,** aging for 14 cycles (illumination for 168 h) in the day/night cycling working mode.

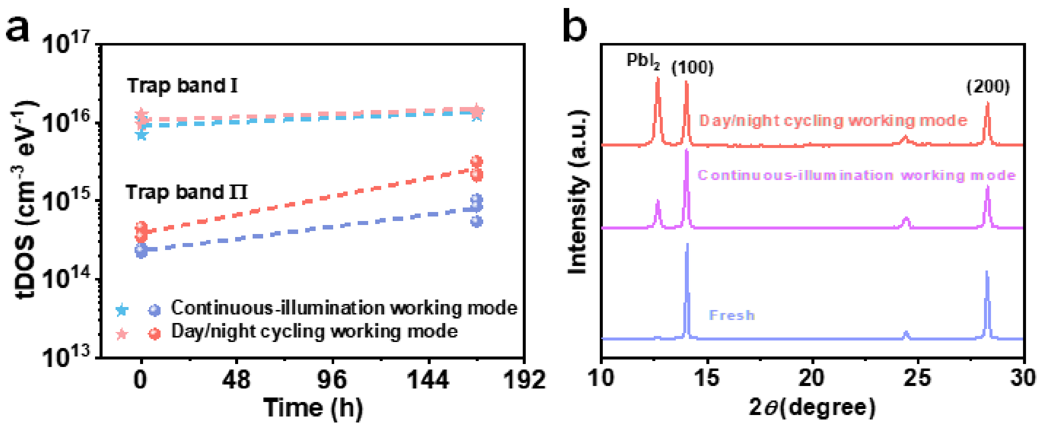

Supplementary Fig. 12 | Defect-evolution dynamics. a, The trap density of states (tDOS) of trap bands I and II for the $FAPbI_3$ based pero-SCs before and after aging in the continuous-illumination (168 h) and day/night cycling (14 cycles) working modes. **b,** X-ray diffraction (XRD) patterns of perovskite films based on $FAPbI_3$ after aging for 168 h in the continuous-illumination and day/night cycling (14 cycles) working modes. The aged perovskite films were prepared by pressing a 3MTemfle × 1700 adhesive tape onto aged devices and separating the electrode from films, where chlorobenzene was used to wash off the hole transport layer from the films.

In pure iodide perovskites, trap band I and II are usually referred to negative (I_i^-) and positive (I_i^+) iodide interstitials, respectively.[6] These results indicate that I_i^+ deep traps are more likely to form within the perovskite under the cycling condition, possibly due to the trapping of photo-induced holes (h^+) by the shallow I_i^- ($I_i^- + h^+ \rightarrow I_i^0$ and $I_i^0 + h^+ \rightarrow I_i^+$), followed by the degradation reactions: $I_i^- + I_i^+ \rightarrow I_2$ and leaving lead cluster (Pb^0).[7]

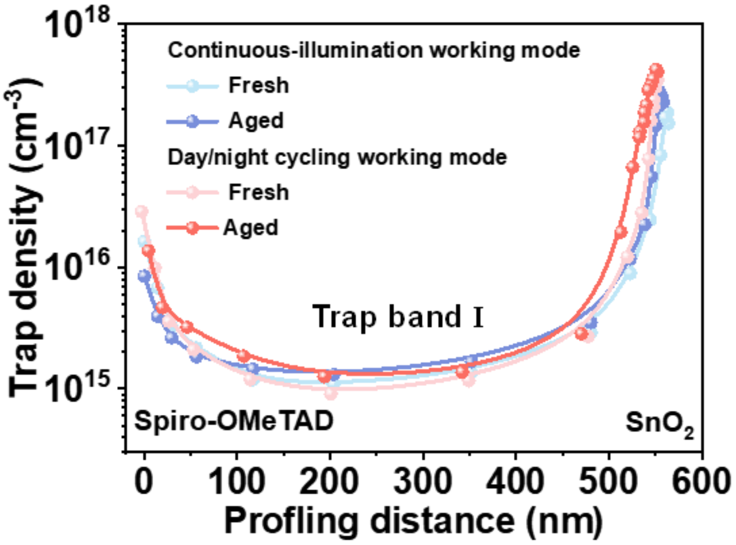

Supplementary Fig. 13 | Defect-evolution dynamics. Spatial distribution of the trap densities of trap band I in the pero-SCs before and after aging in the continuous-illumination (168 h) and day/night cycling (14 cycles) working modes. “SnO_2” and “Spiro-OMeTAD” in the graphs indicate the locations that are close to the SnO_2 or Spiro-OMeTAD layers of the device.

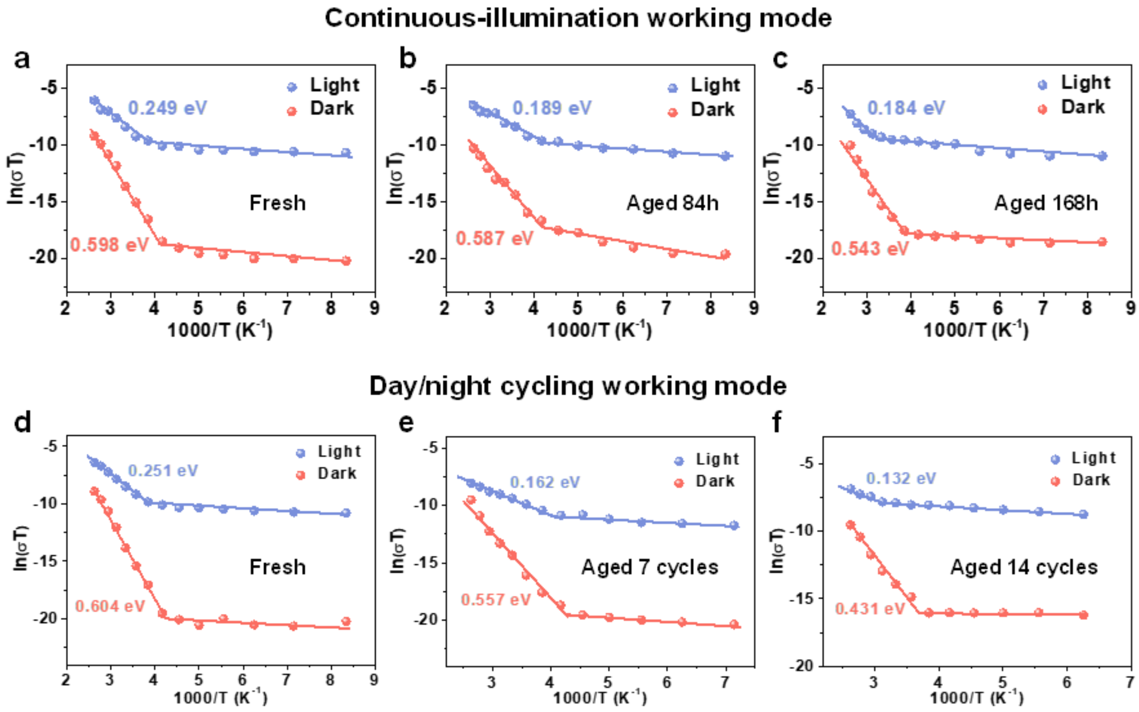

Supplementary Fig. 14 | Ion-migration dynamics. a-c, Temperature-dependent conductivity of the $FAPbI_3$ device **a,** before and after aging for **b,** 84 h and **c,** 168 h in the continuous-illumination working mode. **d-f,** Temperature-dependent conductivity of the $FAPbI_3$ device **d,** before and after aging for 7 cycles (illumination for 84 h, **e**) and 14 cycles (illumination for 168 h, **f**) in the day/night cycling

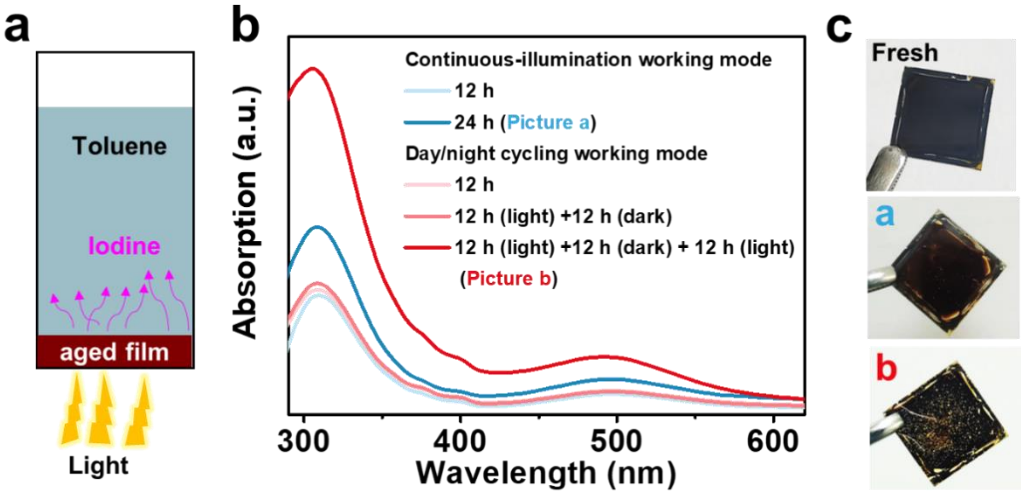

Supplementary Fig. 15 | Iodine-loss analysis of $FAPbI_3$ films. a, Schematic of the iodine-loss experimental setup: vials filled and sealed in N_2, containing $FAPbI_3$ films fully submerged in toluene, were exposed to 1 sun illumination at ~55°C. **b,** Ultraviolet-visible (UV-vis) absorption spectra of the toluene in which $FAPbI_3$ films were immersed in the continuous-illumination and day/night cycling working modes. **c,** Corresponding pictures of the aged $FAPbI_3$ films.

We measured the amount of the yielded I_2 by completely submerging $FAPbI_3$ in toluene. As shown in Supplementary Fig. 15b, the toluene in contact with the day/night-cycling-aged $FAPbI_3$ shows a much stronger absorption signal of I_2 (located at ~500 nm) than that obtained from the $FAPbI_3$ operating in the continuous mode, confirming that the cycling $FAPbI_3$ is more likely to decompose.

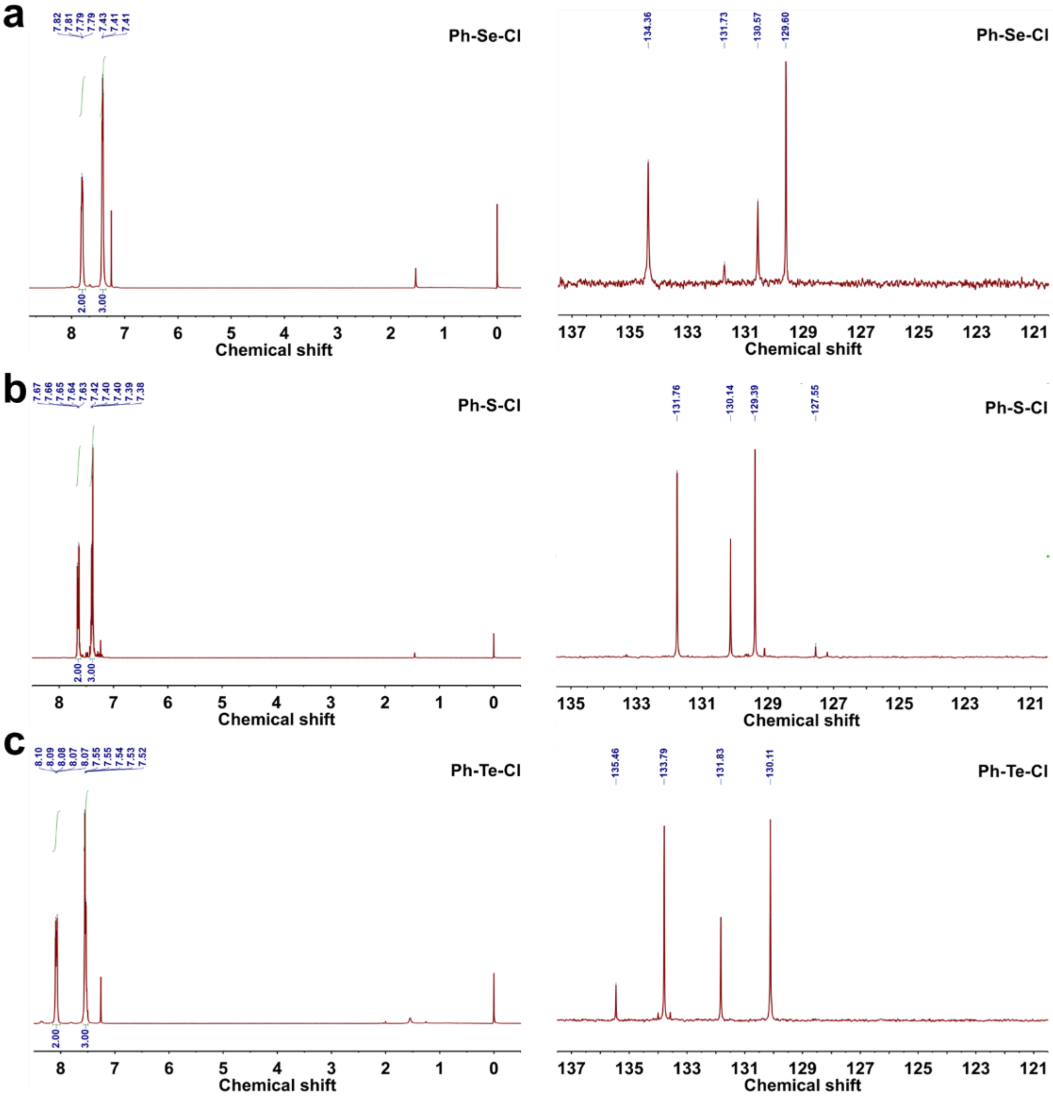

Supplementary Fig. 16 | Different chalcogenides additives. a-c, ^{1}H NMR spectrums and ^{13}C NMR spectrums of **a,** Ph-Se-Cl, **b,** Ph-S-Cl and **c,** Ph-Te-Cl.

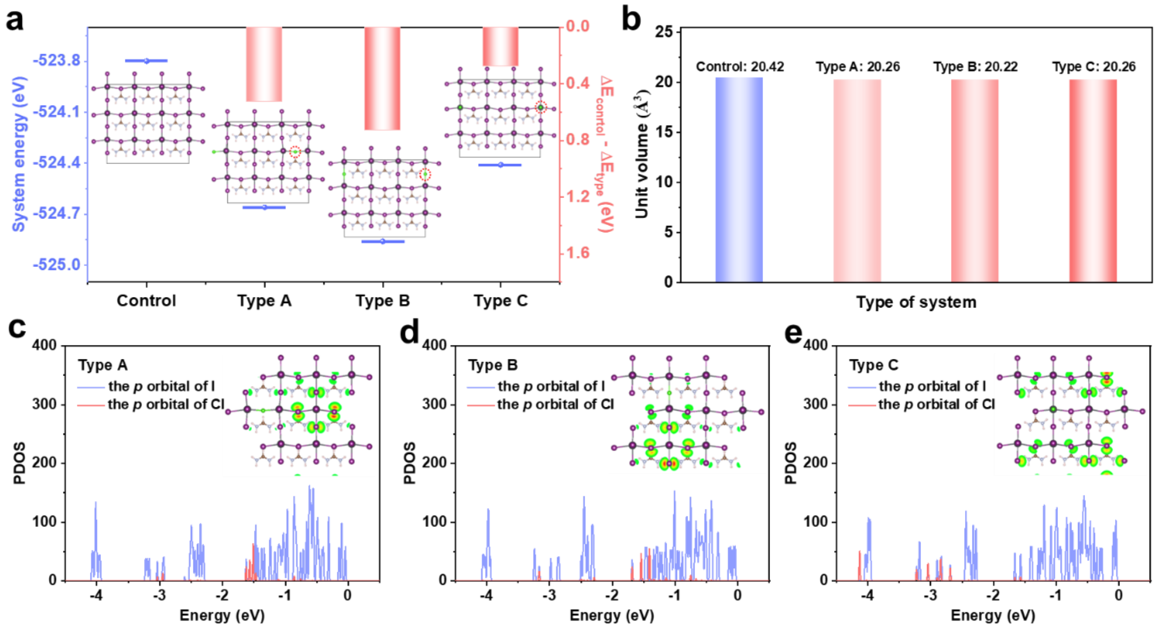

Supplementary Fig. 17 | Theoretical calculation for the role of Cl on the α-$FAPbI_3$ perovskite structure. a, System energies (left) and formation energy difference (right, $\Delta E_{systm} - \Delta E_{type} = E_{system(control)} - E_{system(type)} - E_{I} + E_{Cl}$) between α-$FAPbI_3$ perovskite structure and three possible types of lattice structure with Cl. **b,** Unit volume of α-$FAPbI_3$ perovskite structure and that with Cl. **c-e,** Projected density of states (PDOS) and highest-occupied molecular orbitals (HOMOs) of α-$FAPbI_3$ perovskite with Cl.

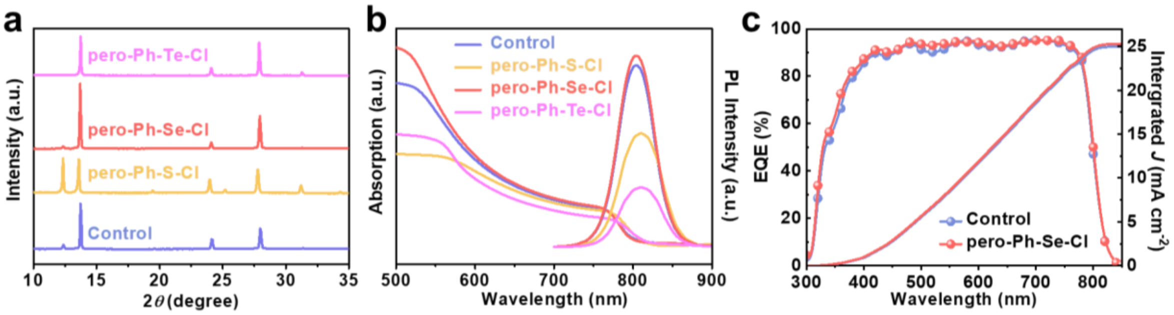

Supplementary Fig. 18 | The performance of perovskite films with different chalcogenides. **a,** XRD patterns of the $FAPbI_3$ perovskite films based on different chalcogenides. **b,** UV–vis absorption spectra and steady-state PL spectra of perovskite films based on different chalcogenides. **c,** External quantum efficiency (EQE) spectrum of the pero-SCs.

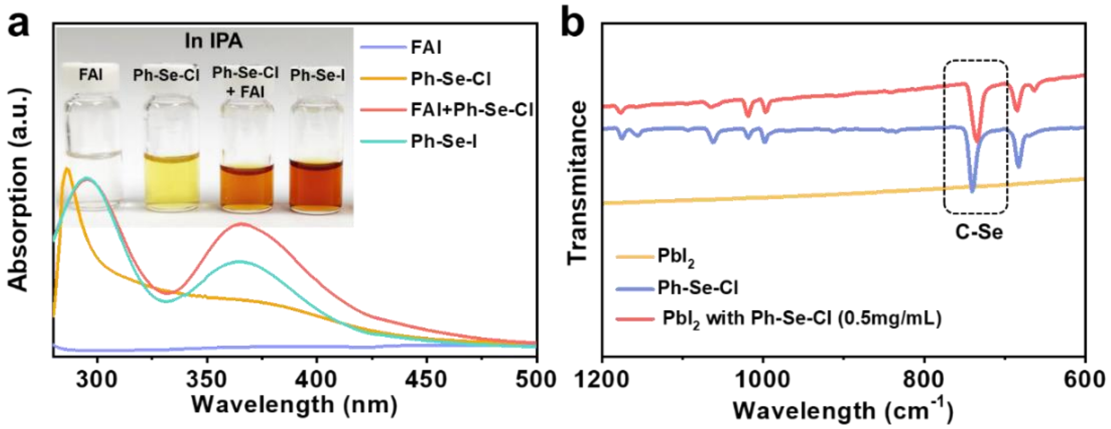

Supplementary Fig. 19 | Interactions between Ph-Se-Cl and perovskite precursors. a, UV–vis absorption spectra of FAI, Ph-Se-Cl, Ph-Se-Cl+FAI and Ph-Se-I in IPA solution. Insert: pictures of the corresponding solutions. **b,** Fourier transform infrared (FTIR) spectra of the PbI_2, PbI_2 mixing Ph-Se-Cl, and Ph-Se-Cl powder.

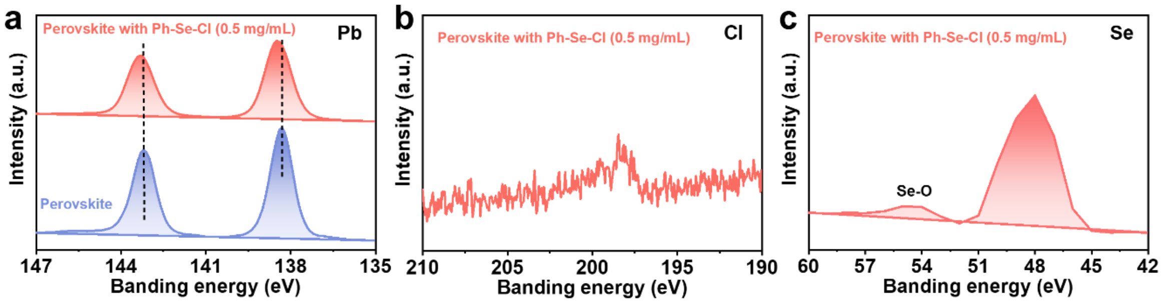

Supplementary Fig. 20 | Interactions between Ph-Se-Cl and perovskite precursors. a-c, X-ray photoelectron spectroscopy (XPS) analyses for the perovskite and perovskite containing Ph-Se-Cl films.

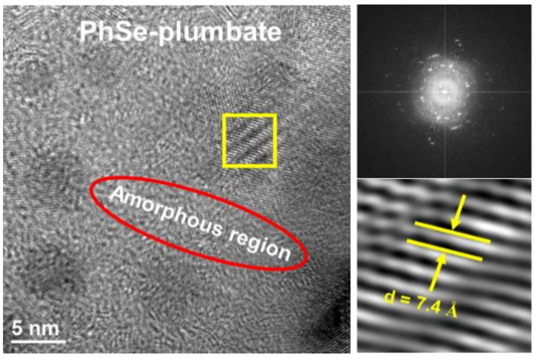

Supplementary Fig. 21 | The formation of PhSe-plumbate. HR-TEM images of the PhSe-plumbate powders. Inset pictures show Fast Fourier transform (FFT) analysis of representative area. The evaluated inter-planar spacing distance of 7.4 Å corresponds to the reflection peak of PhSe-plumbate.

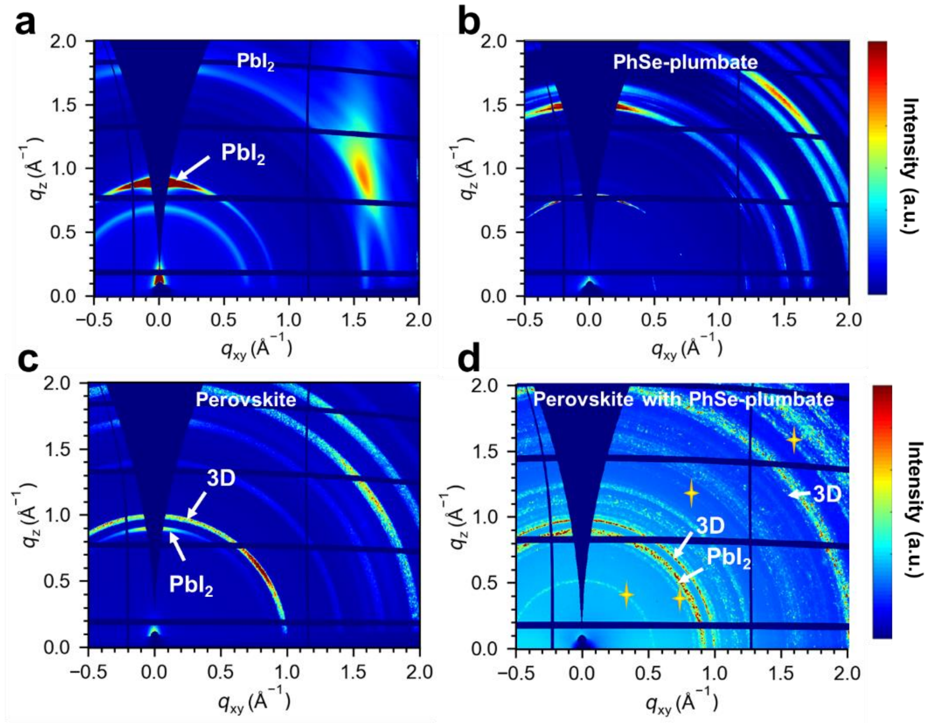

Supplementary Fig. 22 | The formation of PhSe-plumbate. a-d, GIWAXS maps of the **a,** PbI_2 film, **b**, PhSe-plumbate film, **c,** perovskite film and **d,** perovskite film with PhSe-plumbate. (Star signs representing the characteristic peaks of PhSe-plumbate). Note: as for the perovskite film with PhSe-plumbate, 20 mg mL^{-1} Ph-Se-Cl was dissolved in ammonium salt solution in the two-step method for a more obvious diffraction signal.

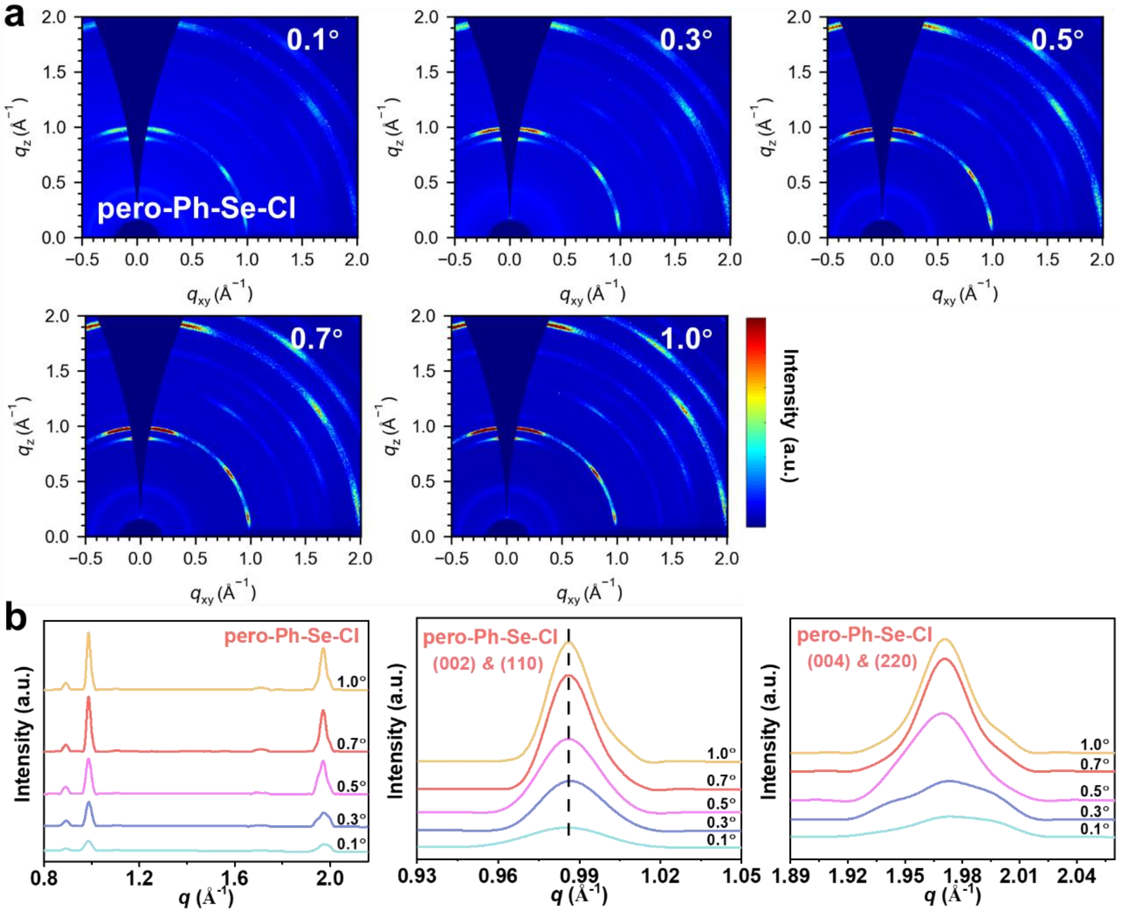

Supplementary Fig. 23 | The pseudo-cubic phase for pero-Ph-Se-Cl. a, The incidence angle-dependent GIWAXS maps of the pero-Ph-Se-Cl film with different incidence angles (0.1°, 0.3°, 0.5°, 0.7° and 1.0°). **b,** Integrated profiles obtained from the GIWAXS maps for the pero-Ph-Se-Cl film with different incidence angles.

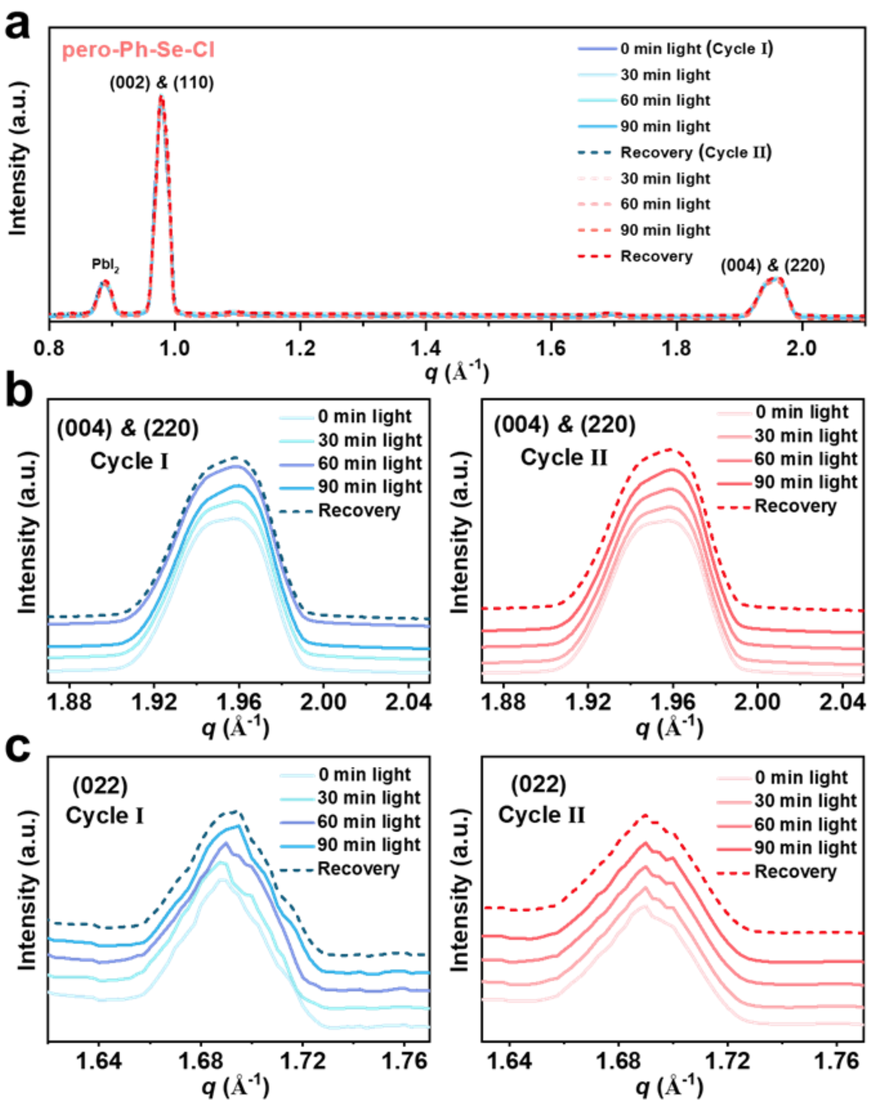

Supplementary Fig. 24 | The pseudo-cubic phase for pero-Ph-Se-Cl. a-c, Integrated profiles obtained from the *in-situ* GIWAXS maps for the pero-Ph-Se-Cl film under illumination from 0 to 90 min, measured at 30-min intervals for the two cycles, and the recovery spectra obtained from the film kept in dark for 30 min (focus on (004)/(220) and (022)).

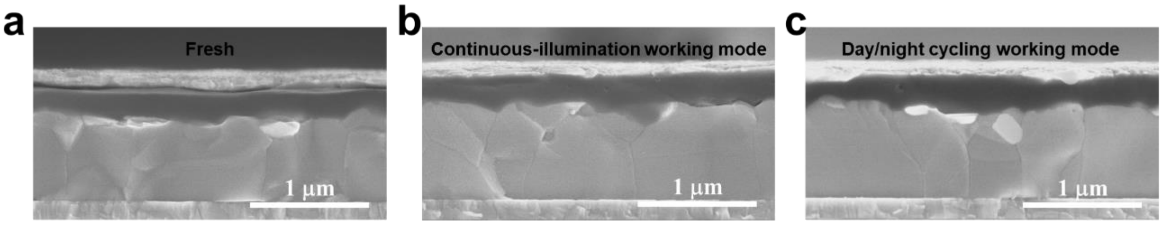

Supplementary Fig. 25 | The degradation of pero-Ph-Se-Cl. a-c, Cross-sectional SEM images of pero-SCs based on pero-Ph-Se-Cl before and after aging in the continuous-illumination (156 h) and day/night cycling (13 cycles) working modes.

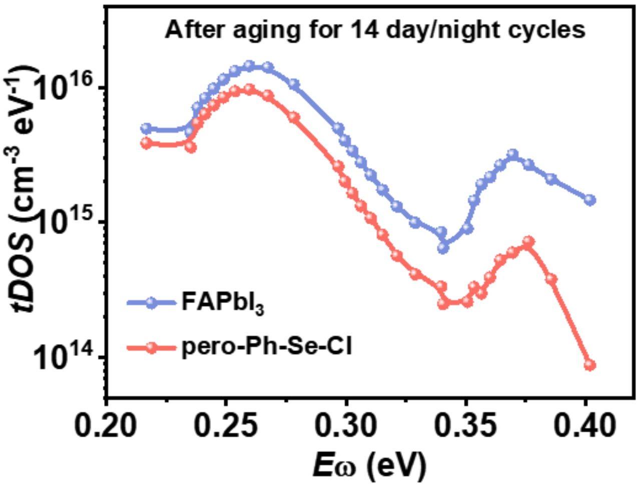

Supplementary Fig. 26 | Decreased defect by Ph-Se-Cl. The tDOS of the $FAPbI_3$-based and pero-Ph-Se-Cl-based devices after aging for 14 cycles in the day/night cycling working mode.

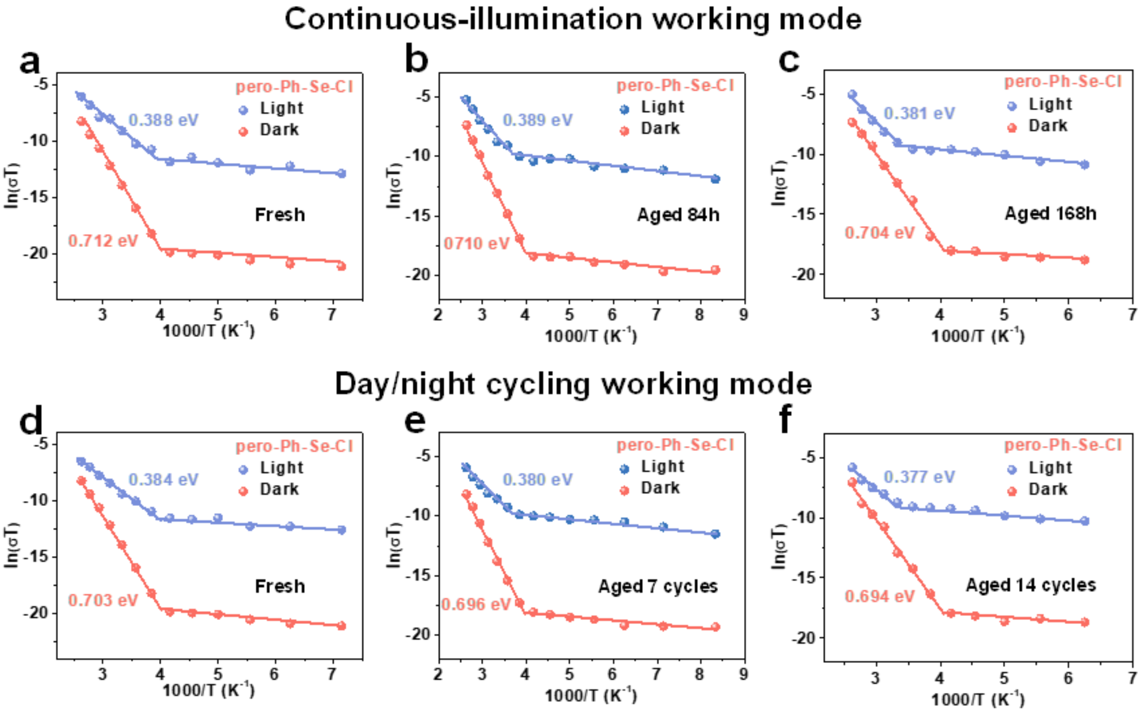

Supplementary Fig. 27 | Minimized the ion migration by Ph-Se-Cl. a-c, Temperature-dependent conductivity of the pero-Ph-Se-Cl-based device **a,** before and after aging for **b,** 84 h and **c,** 168 h in the continuous-illumination working mode. **d-f,** Temperature-dependent conductivity of the pero-Ph-Se-Cl-based device **d**, before and after aging for 7 cycles (illumination for 84 h, **e**) and 14 cycles (illumination for 168 h, **f**) in the day/night cycling working mode.

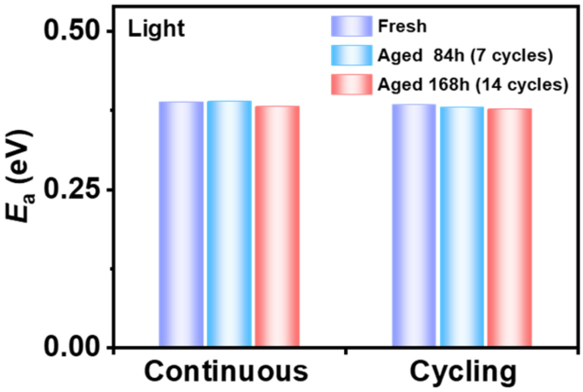

Supplementary Fig. 28 | Minimized the ion migration by Ph-Se-Cl. E_a of the pero-Ph-Se-Cl-based devices before and after aging in the continuous-illumination and day/night cycling working modes.

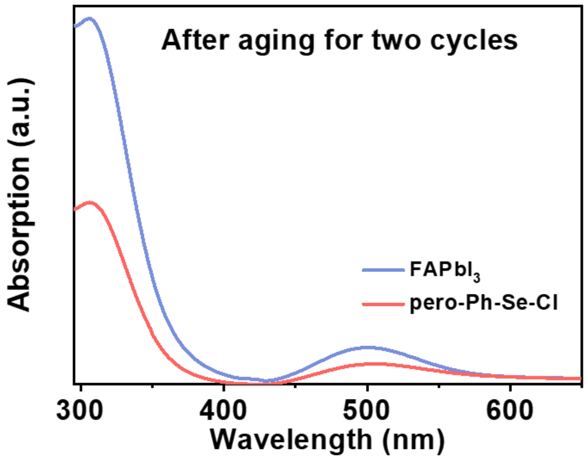

Supplementary Fig. 29 | Minimized the ion migration by Ph-Se-Cl. Absorbance of iodine extracted from the perovskite films immersed in toluene after aging for two cycles in the day/night cycling working mode.

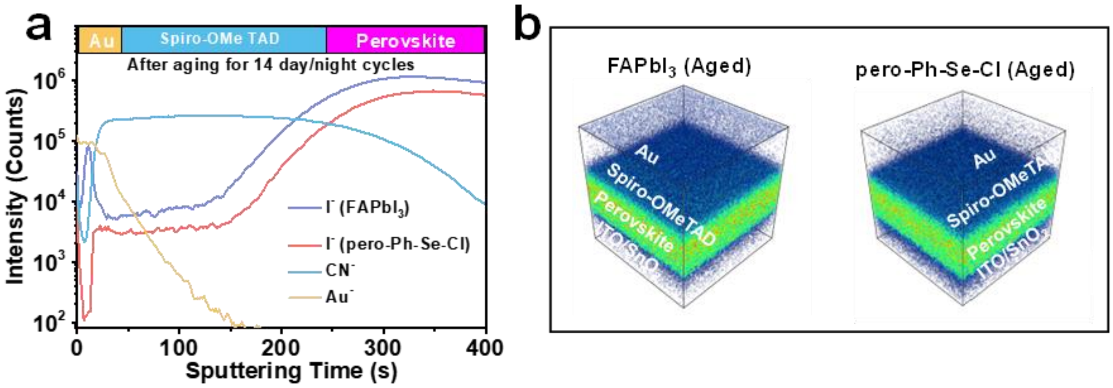

Supplementary Fig. 30 | Minimized the ion migration by Ph-Se-Cl. a, ToF-SIMS depth profiles of the $FAPbI_3$-based and pero-Ph-Se-Cl-based devices after aging for 14 cycles in the day/night cycling working mode. **b,** Reconstructed elemental 3D maps for element I^-.

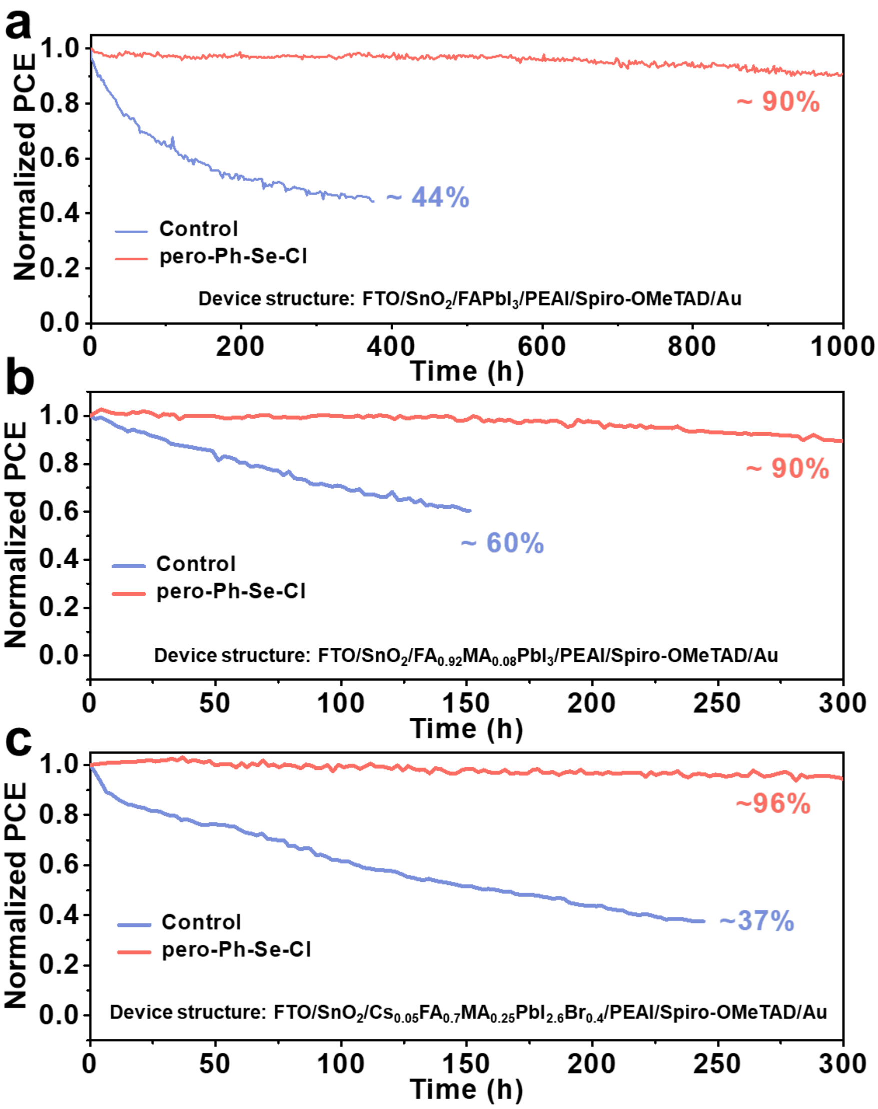

Supplementary Fig. 31 | Stability of the pero-Ph-Se-Cl-based devices. a-c, PCE tracking of the pero-SCs based on **a,** $FAPbI_3$; **b,** $FA_{0.92}MA_{0.08}PbI_3$ and **c,** $Cs_{0.05}FA_{0.7}MA_{0.25}PbI_{2.6}Br_{0.4}$ in the continuous-illumination working mode.

SIMIT

MA 230017163974 ilac-MRA CNAS 中国认可 国际互认 检测 TESTING CNAS L8490

Test and Calibration Center of New Energy Device and Module,
Shanghai Institute of Microsystem and Information Technology,
Chinese Academy of Sciences (SIMIT)

Measurement Report

Report No. 23TR101201

**Client Name**	Soochow University
**Client Address**	199 Renai Road, Industrial Park, Suzhou, Jiangsu, China
**Sample**	Perovskite solar cell
**Manufacturer**	Soochow University
**Measurement Date**	12th October, 2023

**Performed by:**	Qiang Shi *Qiang Shi*	**Date:** 12/10/2023
**Reviewed by:**	Wenjie Zhao *Wenjie Zhao*	**Date:** 12/10/2023
**Approved by:**	Yucheng Liu *Yucheng Liu*	**Date:** 12/10/2023

**Address:** No.235 Chengbei Road, Jiading, Shanghai	**Post Code:**201800
**E-mail:** solarcell@mail.sim.ac.cn	**Tel:** +86-021-69976921

The measurement report without signature and seal are not valid.
This report shall not be reproduced, except in full, without the approval of SIMIT.

1 / 3

SIMIT Report No. 23TR101201

Sample Information

Sample Type	Perovskite solar cell
Serial No.	66-2#
Lab Internal No.	23101201-1#
Measurement Item	I-V characteristic
Measurement Environment	23.8±2.0°C,41.8±5.0%R.H

Measurement of I-V characteristic

**Reference cell**	PVM 1121
**Reference cell Type**	mono-Si, WPVS, calibrated by NREL (Certificate No. ISO 2075)
**Calibration Value/Date of Calibration for Reference cell**	144.53mA/ Feb. 2023
**Measurement Conditions**	Standard Test Condition (STC):<br>Spectral Distribution: AM1.5 according to IEC 60904-3 Ed.3,<br>Irradiance: 1000±50W/m², Temperature: 25±2°C
**Measurement Equipment/ Date of Calibration**	AAA Steady State Solar Simulator (YSS-T155-2M) / July.2023<br>IV test system (ADCMT 6246) / June. 2023<br>SR Measurement system (CEP-25ML-CAS) / April.2023<br>Measuring Microscope (MF-B2017C) / July.2023
**Measurement Method**	I-V Measurement:<br>Logarithmic sweep in both directions (Isc to Voc and Voc to Isc) during one flash based on IEC 60904-1:2020;<br>Spectral Mismatch factor was calculated according to IEC 60904-7 and I-V correction according to IEC 60891;
**Measurement Uncertainty**	Area: 1.0%(k=2); Isc: 1.9%(k=2); Voc: 1.0%(k=2);<br>Pmax: 2.3%(k=2); Eff: 2.5%(k=2)

2 / 3

SIMIT Report No. 23TR101201

====Measurement Results ====

	Forward Scan (Isc to Voc)		Reverse Scan (Voc to Isc)	
Area	5.83 mm²			
Isc	1.530	mA	1.529	mA
Voc	1.186	V	1.187	V
Pmax	1.499	mW	1.535	mW
Ipm	1.451	mA	1.459	mA
Vpm	1.033	V	1.052	V
FF	82.66	%	84.55	%
Eff	25.72	%	26.32	%

- Spectral Mismatch Factor: SMM=0.9950.
- Designated illumination area defined by a thin metal mask was measured by measuring microscope.
- Sample was placed on a thermoelectric cooling stage with a temperature below 10°C. The temperature of the sample was not monitored during the test.
- Test results listed in this measurement report refer exclusively to the mentioned measured sample.
- The results apply only at the time of the test, and do not imply future performance.

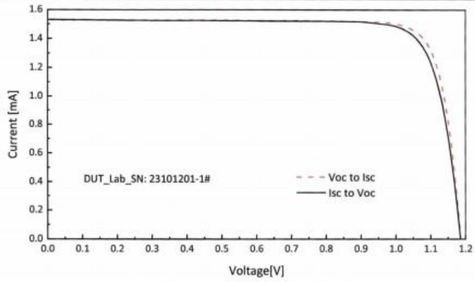

Fig.1 I-V curves of the measured sample

-------End of Report-------

3 / 3

Supplementary Fig. 32 | Certificated results for pero-Ph-Se-Cl-based pero-SCs by SIMIT in Shanghai, China. The certificated efficiency is 26.3%. A mask with an area of 0.0583 cm^2 was used.

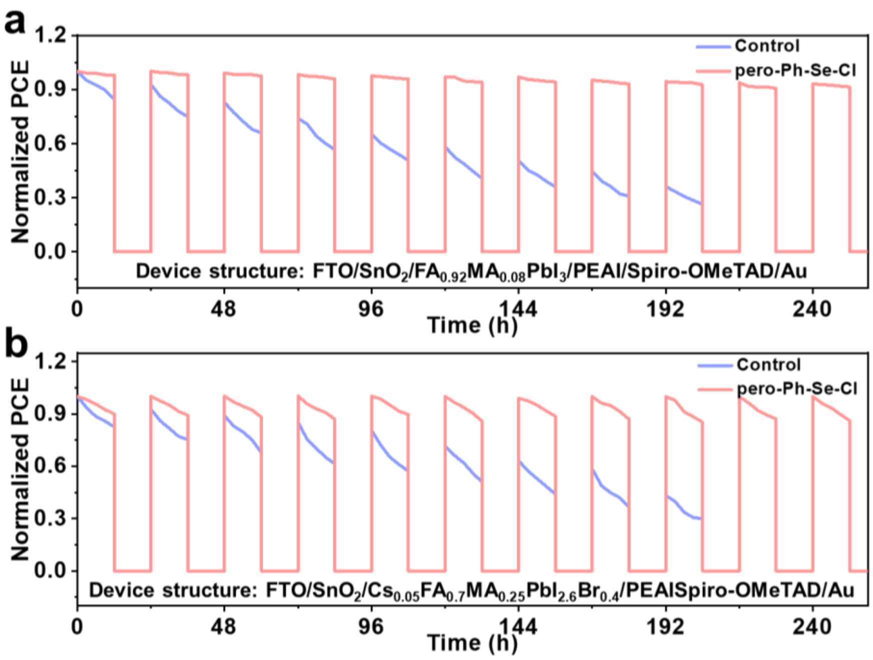

Supplementary Fig. 33 | Stability of the pero-Ph-Se-Cl-based devices. a,b, PCE tracking of the pero-SCs without and with Ph-Se-Cl modification based on **a,** $FA_{0.92}MA_{0.08}PbI_3$ and **b,** $Cs_{0.05}FA_{0.7}MA_{0.25}PbI_{2.6}Br_{0.4}$ in the day/night cycling working mode.

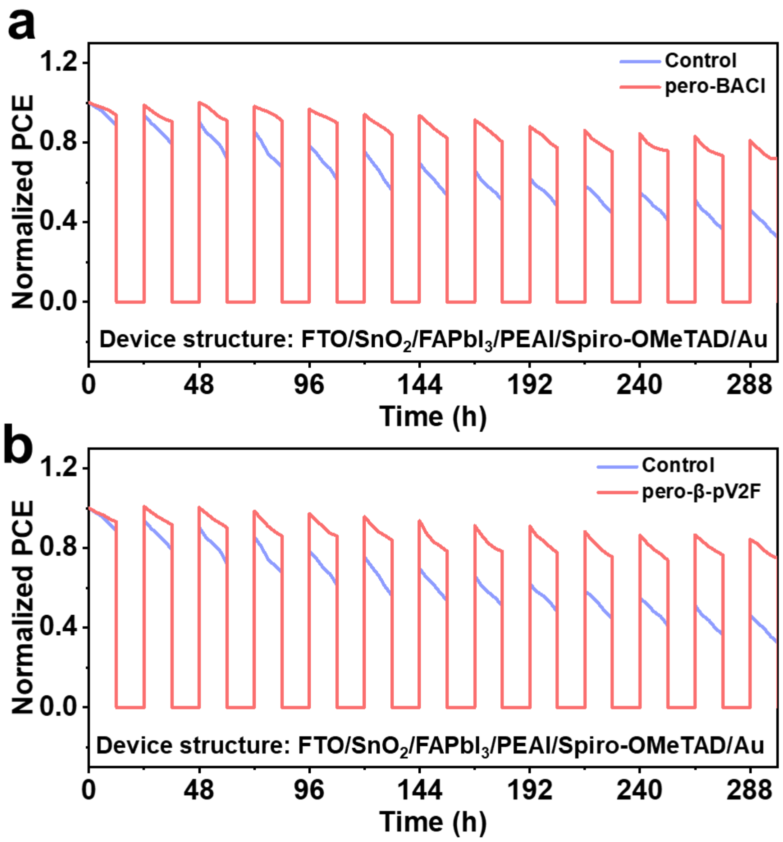

Supplementary Fig. 34 | Stability of the devices. a,b, The stability of the pero-SCs with **a,** BACl and **b,** β-pV2F as additives in the day/night cycling working mode.

Supplementary Tables

Supplementary Table 1 | The review of device structure and test protocols in day/night cycling operation stability studies on pero-SCs.

Device structure	PCE	Test details	Ref.
FTO/TiO_2/$MAPbI_3$/Spiro-OMeTAD/Au	13.8%	solar simulator<br>1. 12-h light on/off cycles **at 25°C** (test 84h)<br>2. 12-h dark (-10°C) and 12-h light (**25°C**) cycles (test 324h)	Nano Energy, 27, 509-514 (2016)[8]
a. FTO/c-TiO_2/$MAPbI_3$ /Spiro-OMeTAD/Au<br>b. FTO/c-TiO_2/m-TiO_2/ $MAPbI_3$ /Spiro-OMeTAD/Au<br>c. FTO/c-NiO_x/$MAPbI_3$/ PCBM/BCP/Ag	a. 17.2%<br>b. 18.3%<br>c. 15.3%	solar simulator<br>12-h light on/off cycles **at 25°C** (test 240h)	Nano Energy, 58, 687-694 (2019)[9]
FTO/TiO_2/$MAPbI_xBr_{3-x}$/Spiro-OMeTAD/Au	18.8%-20.7%	UV-filtered one Sun equivalent light<br>MPP tracking for device was stopped after only 5 h and repeated periodically after leaving the device resting in the dark for a varying number of hours (test 100h)	Energy Environ. Sci., 10, 604, (2017)[10]
FTO/ c-In-TiO_2/m-TiO_2/ PMMA:PCBM/$Cs_{0.07}Rb_{0.03}FA_{0.765}MA_{0.135}PbI_{2.55}Br_{0.45}$/Spiro-OMeTAD/Au	20.4%	MPP tracking<br>12-h light on/off cycles **at 25°C** (test 84h)	Energy Environ. Sci., 10, 1792, (2017)[11]
ITO/PEDOT:PSS/$MAPbI_3$ /PCBM/Al	~15%	MPP tracking for device was stopped after only 2 h and repeated periodically after leaving the device resting in the dark for a varying number of hours (test 24h)	Nat. Commun. 7, 11574 (2016)[12]
FTO/c-TiO_2/m-TiO_2/ $FA_{0.83}MA_{0.17}Pb(I_{0.83}Br_{0.17})_3$/ Spiro-OMeTAD/Au	>17%	MPP tracking<br>6-h light on/off cycles **at 20°C** (test 276h)	Nat. Energy 3, 61-67 (2018)[13]
FTO/SnO_2/$FAPbI_3$/PEAI/Spiro-OMeTAD/Au	26.3%	solar simulator<br>MPP tracking<br>12-h dark (RT) and 12-h light (~55°C) cycles (test over 1000h)	This work

Supplementary Table 2 | The initial performance of all devices used in the study.

Device structure		V_{oc} (V)	J_{sc} (mA cm^{-2})	FF (%)	PCE (%)
$FTO/SnO_2/FAPbI_3/PEAI/Spiro-OMeTAD/Au$	Control	1.150 ± 0.012	25.89 ± 0.91	80.68 ± 1.72	24.02 ± 0.48
	Pero-Ph-Se-Cl	1.181 ± 0.011	26.23 ± 0.82	83.95 ± 1.05	26.01 ± 0.31
$FTO/SnO_2/FA_{0.92}MA_{0.08}PbI_3/PEAI/Spiro-OMeTAD/Au$	Control	1.141 ± 0.021	24.98 ± 1.05	79.26 ± 1.19	22.59 ± 0.69
	Pero-Ph-Se-Cl	1.162 ± 0.014	25.37 ± 1.34	82.83 ± 1.37	24.42 ± 0.51
$FTO/SnO_2/Cs_{0.05}FA_{0.7}MA_{0.25}PbI_{2.6}Br_{0.4}/PEAI/Spiro-OMeTAD/Au$	Control	1.182 ± 0.023	21.81 ± 1.27	77.56 ± 1.14	20.00 ± 0.74
	Pero-Ph-Se-Cl	1.201 ± 0.012	22.99 ± 1.01	79.85 ± 1.23	22.05 ± 0.64
$FTO/SnO_2/FAPbI_3/BDT-DPA-F/Au$	Control	1.115 ± 0.026	25.10 ± 1.27	73.21 ± 1.16	20.48 ± 0.50
	Pero-Ph-Se-Cl	1.134 ± 0.018	25.34 ± 1.22	77.64 ± 1.21	22.31 ± 0.34
$FTO/SnO_2/FAPbI_3/OAI/Spiro-OMeTAD/Au$	Control	1.124 ± 0.022	25.80 ± 1.21	80.68 ± 1.48	23.40 ± 0.58
$FTO/SnO_2/FAPbI_3/Spiro-OMeTAD/Au$	Control	1.072 ± 0.032	25.55 ± 1.45	76.24 ± 1.24	20.88 ± 0.84
$FTO/SnO_2/FAPbI_3/$ carbon electrode	Control	1.069 ± 0.017	23.59 ± 1.36	65.74 ± 1.02	16.58 ± 0.44

Supplementary Table 3 | The electronegativity of each atom.

Atom	Electronegativity (Pauling)	Δ |(Pb-X)|	Δ |(I-X)|
I	2.66	0.33	-
Pb	2.33	-	0.33
S	2.58	0.25	0.08
Se	2.55	0.22	0.11
Te	2.12	0.21	0.54

Supplementary Table 4 | Photovoltaic parameters of the devices based on different chalcogenides under AM1.5G illumination (intensity: 100 mW cm^{-2}).

	Concentration	V_{oc} (V)	J_{sc} (mA cm^{-2})	FF (%)	PCE (%)
**Control**	--	1.150 ± 0.012	25.89 ± 0.21	80.68 ± 1.72	24.02 ± 0.48
**pero-Ph-Se-Cl**	0.1 mg mL^{-1}	1.163 ± 0.013	25.95 ± 0.17	81.15 ± 1.55	24.49 ± 0.39
	0.3 mg mL^{-1}	1.171 ± 0.017	26.13 ± 0.15	81.28 ± 1.61	24.87 ± 0.55
	0.5 mg mL^{-1}	1.181 ± 0.011	26.23 ± 0.12	83.95 ± 1.02	26.01 ± 0.31
	0.7 mg mL^{-1}	1.177 ± 0.012	26.13 ± 0.11	82.76 ± 1.25	25.45 ± 0.43
**pero-Ph-S-Cl**	0.1 mg mL^{-1}	1.041 ± 0.021	24.55 ± 0.34	72.16 ± 1.45	18.44 ± 0.63
	0.3 mg mL^{-1}	1.045 ± 0.019	24.63 ± 0.28	73.30 ± 1.32	18.87 ± 0.53
	0.5 mg mL^{-1}	1.051 ± 0.011	24.64 ± 0.27	73.28 ± 1.76	18.98 ± 0.51
	0.7 mg mL^{-1}	1.000 ± 0.018	24.42 ± 0.31	72.32 ± 1.33	17.66 ± 0.47
**pero-Ph-Te-Cl**	0.1 mg mL-1	1.127 ± 0.016	24.45 ± 0.21	67.30 ± 1.03	18.54 ± 0.48
	0.3 mg mL-1	1.130 ± 0.014	24.61 ± 0.24	66.71 ± 1.29	18.55 ± 0.42
	0.5 mg mL-1	1.129 ± 0.013	24.66 ± 0.20	67.75 ± 1.14	18.86 ± 0.46
	0.7 mg mL-1	1.103 ± 0.015	24.21 ± 0.26	65.21 ± 1.22	17.41 ± 0.45

Supplementary Table 5 | The normalized lattice parameters refinement the *in-situ* GIWAXS data with the Le Bail method.[14]

	**Nor. a**	**Nor. b**	**Nor. c**	**a_0**	**Nor. Vol.**	**ε_{tet}**	**ε_{orth}**
**Control**	6.387	6.532	6.544	6.487	273.042	0.015	0.022
**pero-Ph-Se-Cl**	6.427	6.535	6.544	6.502	274.839	0.011	0.017

Here the approximate changes to the lattice parameters are made relative to the cubic unit cell and are used to normalize our consideration of the lower symmetry phases. Parameters ε_{tet} and ε_{orth} respectively represent the degenerate tetragonal and orthorhombic symmetry-adapted strain which emerge during the phase transitions. Relative to an undistorted cubic (a_0; estimated by taking the cube root of the normalized unit cell volume), the strain components are calculated by $\varepsilon_1 = (a - a_0)/a_0$, $\varepsilon_2 = (b - a_0)/a_0$, and $\varepsilon_3 = (c - a_0)/a_0$, where a, b, and c are the normalized lattice parameters of the perovskite. Thus, the symmetry-adapted strains driving the phase sequence above are defined by $\varepsilon_{tet} = 1/3^{1/2}\,(2\varepsilon_3 - \varepsilon_2 - \varepsilon_1)$; $\varepsilon_{orth} = \varepsilon_2 - \varepsilon_1$. The factor of $3^{1/2}$ is introduced to ensure that the two strains appear on the same scale.

Supplementary Table 6 | Photovoltaic parameters of the champion devices under AM1.5G illumination (intensity: 100 mW cm^{-2}).

	Scan direction	V_{oc} (V)	J_{sc} (mA cm^{-2})	FF (%)	PCE (%)
**Control**	Reverse	1.160	25.99	81.28	24.50
	Forward	1.151	25.91	78.64	23.45
**pero-Ph-Se-Cl**	Reverse	1.187	26.23	84.55	26.32
	Forward	1.186	26.23	82.66	25.72

Additional references

1. Wang, X. et al. Long-chain anionic surfactants enabling stable perovskite/silicon tandems with greatly suppressed stress corrosion. *Nat. Commun.* **14**, 2166 (2023).
2. Chason, E. & Guduru, P. R. Tutorial: Understanding residual stress in polycrystalline thin films through real-time measurements and physical models. *J. Appl. Phys.* **119**, 191101 (2016).
3. Hopcroft, M. A., Nix, W. D. & Kenny, T. W. What is the Young's Modulus of Silicon? *J. Microelectromech. S.* **19**, 229-238 (2010).
4. Kim, M. et al. Methylammonium chloride induces intermediate phase stabilization for efficient perovskite solar cells. *Joule* **3**, 2179-2192 (2019).
5. Fabini, D. H. et al. Reentrant structural and optical properties and large positive thermal expansion in perovskite formamidinium lead iodide. *Angew. Chem. Int. Ed.* **55**, 15392-15396 (2016).
6. Ni, Z. et al. Evolution of defects during the degradation of metal halide perovskite solar cells under reverse bias and illumination. *Nat. Energy* **7**, 65-73 (2022).
7. Motti, S. G. et al. Controlling competing photochemical reactions stabilizes perovskite solar cells. *Nat. Photonics* **13**, 532-539 (2019).
8. Huang, F. et al. Fatigue behavior of planar $CH_3NH_3PbI_3$ perovskite solar cells revealed by light on/off diurnal cycling. *Nano Energy* **27**, 509-514 (2016).
9. Jiang, L. et al. Fatigue stability of $CH_3NH_3PbI_3$ based perovskite solar cells in day/night cycling. *Nano Energy* **58**, 687-694 (2019).
10. Domanski, K. et al. Migration of cations induces reversible performance losses over day/night cycling in perovskite solar cells. *Energy Environ. Sci.* **10**, 604-613 (2017).
11. Peng, J. et al. Interface passivation using ultrathin polymer–fullerene films for high-efficiency perovskite solar cells with negligible hysteresis. *Energy Environ. Sci.* **10**, 1792-1800 (2017).
12. Nie, W. et al. Light-activated photocurrent degradation and self-healing in perovskite solar cells. *Nat. Commun.* **7**, 11574 (2016).
13. Domanski, K., Alharbi, E. A., Hagfeldt, A., Grätzel, M. & Tress, W. Systematic investigation of the impact of operation conditions on the degradation behaviour of perovskite solar cells. *Nat. Energy* **3**, 61-67 (2018).
14. Steele, J. A. et al. Trojans that flip the black phase: Impurity-driven stabilization and spontaneous strain suppression in γ-$CsPbI_3$ perovskite. *J. Am. Chem. Soc.* **143**, 10500-10508 (2021).
